# Capsid and genome damage are the leading inactivation mechanisms of aerosolized porcine respiratory coronavirus at different relative humidities

**DOI:** 10.1128/aem.02319-24

**Published:** 2025-04-07

**Authors:** Aijia Zhou, P. S. Ganesh Subramanian, Salma El-Naggar, Joanna L. Shisler, Vishal Verma, Thanh H. Nguyen

**Affiliations:** 1 Department of Civil and Environmental Engineering, University of Illinois at Urbana-Champaign, Champaign, Illinois, USA; 2 The Grainger College of Engineering, University of Illinois at Urbana-Champaign 14589, Champaign, Illinois, USA; 3 Department of Bioengineering, University of Illinois at Urbana-Champaign, Champaign, Illinois, USA; 4 Department of Microbiology, University of Illinois at Urbana-Champaign, Champaign, Illinois, USA; 5 Institute of Genomic Biology, University of Illinois at Urbana-Champaign, Champaign, Illinois, USA; 6 Carle Illinois College of Medicine 573544, Urbana, Illinois, USA; Centers for Disease Control and Prevention, Atlanta, Georgia, USA

**Keywords:** RNA virus, coronavirus, aerosols, relative humidity, inactivation mechanisms, RT-qPCR

## Abstract

Relative humidity (RH) varies widely in indoor environments based on temperature, outdoor humidity, heating systems, and other environmental conditions. This study explored how RH affects aerosolized porcine respiratory coronavirus (PRCV), a model for coronaviruses, over a time range from 0 min to a maximum of 1 h, and the molecular mechanism behind viral infectivity reduction. These questions were answered by quantifying: (i) viral-host receptor interactions, (ii) capsid integrity, (iii) viral genome integrity, and (iv) virus infectivity. We found RH did not alter PRCV-receptor interactions. RHs 45–55% and 65–75% damaged viral genomes (2 log_10_ reduction and 1 log_10_ reduction, respectively, in terms of median sample value), whereas RHs 55–65% decreased capsid integrity (2 log_10_ reduction). No apparent virion damage was observed in RH 75–85%. Two assays were used to quantify virus presence: qPCR for detecting the viral genomes and plaque-forming unit assay for detecting the virus replication. Our results indicated that the qPCR assay overestimated the concentrations of infectious viruses, and RNase treatment with long-range RT-qPCR performed better than one-step RT-qPCR. We propose that understanding the influence of RH on the stability of aerosolized viruses provides critical information for detecting and preventing the indoor transmission of coronaviruses.

**IMPORTANCE:**

Indoor environments can impact the stability of respiratory viruses, which can then affect the transmission rates. The mechanisms of how relative humidity (RH) affects virus infectivity still remain unclear. This study found RH inactivates porcine respiratory coronavirus by damaging its capsid and genome. The finding highlights the potential role of controlling indoor RH levels as a strategy to reduce the risk of coronavirus transmission.

## INTRODUCTION

Airborne virus transmission can profoundly impact society. Millions of people died from the COVID-19 pandemic (1), and each year millions of people suffer severe symptoms of influenza virus infection (2). The infections caused by airborne viruses may strain healthcare systems, disrupt daily life, and lead to economic and social upheaval (3, 4). Airborne viruses like SARS-CoV-2 and influenza can be transmitted via aerosols (size <100 µm) (5), droplets, and/or fomites (6), and spread into the populations rapidly in indoor conditions. While larger respiratory droplets dominate SARS-CoV-2 transmission during close-proximity interactions over a shorter timescale (5), aerosol transmission becomes critical in enclosed spaces over larger timescales due to the smaller size of aerosols, which allows viruses to remain suspended in indoor air for extended periods (7, 8). Virus-enriched aerosols (size <5 µm) are heterogeneous when released from infected individuals and contain different virus loads and other components (5). The chances of inhaling viral aerosol and subsequently getting infected could be reduced substantially by factors including relative humidity (RH), temperature, ventilation, and others (9). Appropriate engineering controls of these factors could reduce indoor airborne virus transmission effectively (10–12).

Room temperature is constrained by human adaptation. Some people may prefer higher or lower temperatures than others. Because human behavior is hard to predict, controlling virus spreading by adjusting only indoor temperature is not practical. Previous research has examined the relationships between RH levels and viral aerosol inactivation to better mitigate indoor transmission risk: the inactivation of viral aerosols was found as monotonic (RH has a consistently positive or negative correlation with viral stability), V-shaped (the middle value of a tested RH range has the highest effect on virus stability), or unrelated (RH does not show a significant effect on viral stability) (13–15). The trends found in these few studies were virus specific. Different strains of influenza virus, when aerosolized in the same media, exhibited distinct inactivation trends (16), highlighting the importance of virus-specific factors in viral inactivation, independent of media effects. These studies imply the existence of complicated relationships between RH and airborne viruses. No common trends between RH and viral aerosol infectivity have been reported. For example, the infectivity of aerosolized SARS-CoV-2 increased with RH after 2 min of exposure (17). However, SARS-CoV-2 droplets had the lowest infectivity at RH 65%, compared to RH 40% and RH 85% (18). It is difficult to determine the decay inactivation type for SARS-CoV-2 (e.g., monotonic vs V-shaped) because only a few time points were examined. For other coronaviruses like TGEV (19) and MERS-CoV (20), higher viability of aerosolized viral particles was observed in low RH conditions. More recently, Niazi et al. showed that the RH decay of the H3N2 strain of IAV is not monotonic (14). These observed differences in the relationships between virus infectivity and RH reflect an incomplete understanding of the relationship between RH and virus viability, which in turn complicates the efforts to regulate the indoor heating and ventilation systems so that the transmission of viral aerosol is minimal.

We propose that understanding the mechanisms of viral aerosol inactivation may provide valuable insights for controlling indoor conditions for improved public health. For example, if we know which parts of a virion are destroyed by certain RH levels, this information could be applied to predict the environmental persistence of a new virus within the same family when a new virus starts to cause outbreaks. Consequently, this knowledge might inform strategies for engineering intervention to reduce the virion levels indoors. We conducted a comprehensive study to identify what viral structure, including spike protein, capsid protein, and genome, was damaged using porcine respiratory coronavirus (PRCV) as a model for other similar coronaviruses in aerosols collected under different RH conditions. We believe the results from this study can provide insights into how other coronaviruses, such as SARS-CoV-2, are inactivated at different RH levels.

## RESULTS

### Effects of particle deposition on virus removal after exposure to RH can be quantified

**Fig 1:**
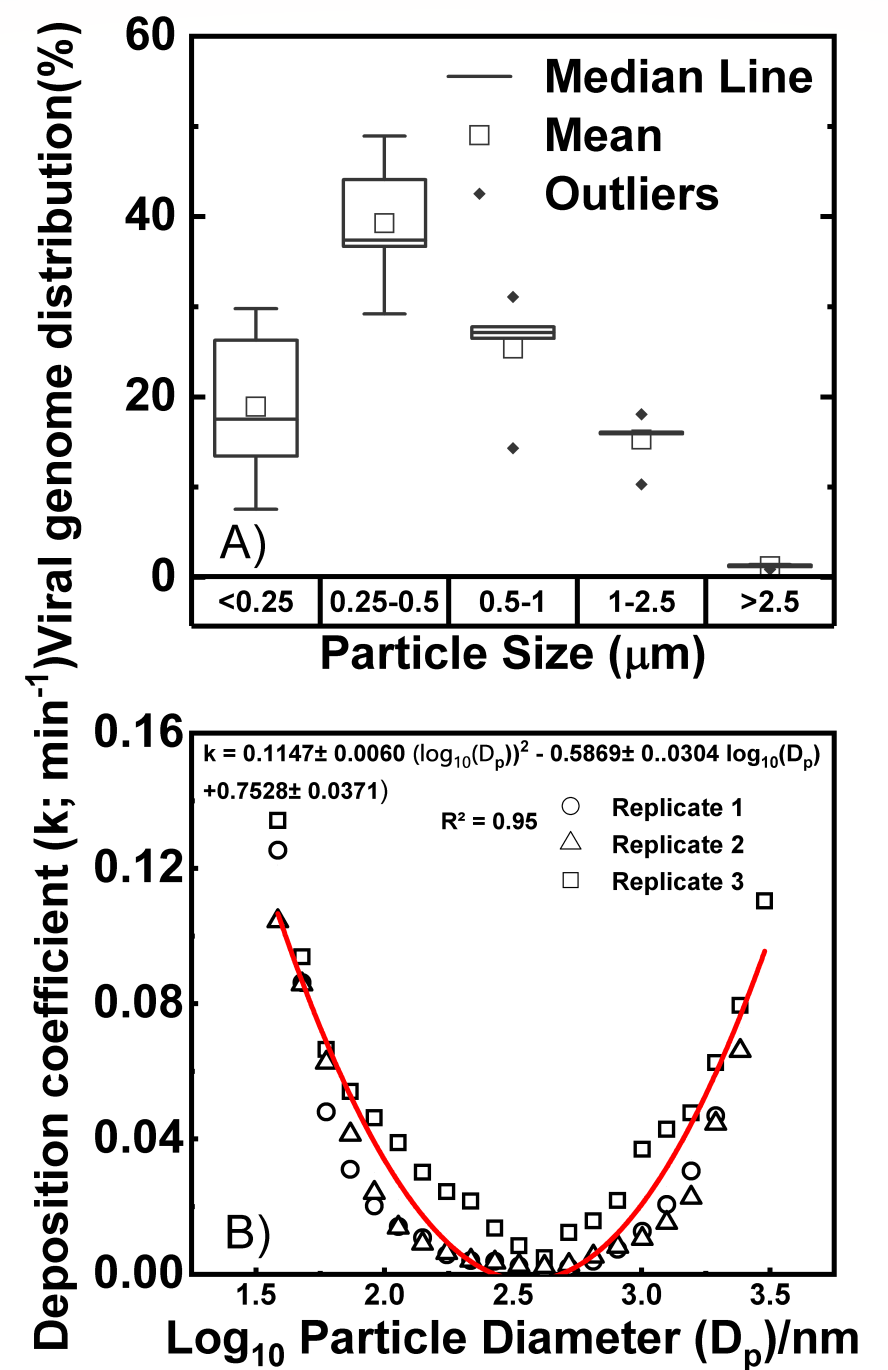
Aerosolized PRCV genome distribution in RH 65–75% as measured by N gene amplicon levels across different particle sizes. (**A**) Viral particle distribution was determined from the fraction collected by the quartz filters inside each stage of the cascade impactor. (**B**) Particle deposition coefficient distribution was determined by combining SMPS and OPS data. See the scatter plot in Fig. S7.

We configured an experimental system that allowed us to examine the PRCV stability under different RH conditions from RH45% to 85% (21). Figure 1A was the example of aerosolized PRCV particle distribution in RH 65–75%. Size distribution in other RHs (Fig. S5) is provided in section VII of the supplemental material. Figure 1A showed that virus-containing particles produced in our system were indeed aerosols, mostly ranging from 0.25 to 2.5 µm in size, similar to the results reported by Niazi et al. (22). We detected these viruses by quantifying the N gene using one-step RT-qPCR and expressed these data as a percentage distribution shown on the *Y*-axis of Fig. 1A. Most of the PRCV genomes were detected in the particle size range of 0.25–0.5 µm, with 0.25–0.5 µm being the most prevalent size range comprising over 40% of the viral genome, followed by 0.5–1 µm. The largest particles over 2.5 µm had the lowest abundance.

Because RH can affect the size and therefore particle deposition rate of aerosolized viruses (23), we determined the relationship between the deposition coefficient (k) and particle diameter ($D_{p}$) in Fig. 1B to calculate particle deposition. The smallest deposition coefficient occurred at the particle diameter of around 10^2.5^ (~320) nm, indicating the lowest particle deposition in this size. These align well with findings from earlier studies which typically report the smallest deposition coefficient occurring for a particle diameter in the range of 200–500 nm (24, 25). From Fig. 1B, we see that the deposition coefficient (k; min^−1^) can be expressed as a function of the particle diameter ($D_{p}$; in nm) using the following equation:


(1) $$\begin{array}{c} k=\left[0.1147\pm 0.0060\right]\cdot \;{\left(\log_{10}D_{p}\right)}^{2}-\left[0.5869\pm 0.0304\right]\cdot \;\left(\log_{10}D_{p}\right)+\left[0.7528\pm 0.0371\right]\; \end{array}$$

The percentage of aerosols suspended in the air (aerosols that are yet to be deposited) at exposure time $(t_{exp}$) can be calculated using the following equation for size *i*:


(2) $$\begin{array}{c} Percentage\;of\;suspended\;aerosols\;S_{i}\left(t_{exp}\right)=100\cdot e^{-k_{i}\cdot t_{exp}} \end{array}$$

The smallest deposition coefficient was 4.3 × 10^−3^ min^−1^; therefore, aerosol loss resulting from leakages was assumed to be negligible based on the low air exchange rate (AER) of the chamber (0.016 h^−1^ = 2.3 × 10^−4^ min^−1^) compared to the deposition loss coefficients. We calculated the percentage of suspended aerosols for different particle sizes and $t_{exp}$ using equation 2. Note that for size ranges <0.25 and >2.5 µm, this percentage is the theoretical maximum that can be reached because only the minimum deposition coefficient value is available (i.e., $k_{0.25\;\mu m}<k_{<0.25\;\mu m}$ and $k_{2.5\;\mu m}<k_{>2.5\;\mu m}$). For other size ranges, the remaining percentage was calculated using the mean values of the percentage of suspended aerosols in the lower and upper bounds of the size interval, as depicted in the following example:


(3) $$\begin{array}{c} S_{0.25-0.5}\;\left(t_{exp}\right)=\frac{S_{0.25}\left(t_{exp}\right)+S_{0.5}\left(t_{exp}\right)}{2} \end{array}$$

See the percentages of suspended aerosols in each size bin for various RH in Tables S4 to S7.

We then calculated the overall remaining percentage $\left[F(t_{exp})\right]$ at $t_{exp}$ for all size ranges of aerosols using the following equation:


(4) $$\begin{array}{c} F\left(t_{exp}\right)=\sum_{j=1}^{5}S_{j}\left(t_{exp}\right)\cdot A_{j}, \end{array}$$

where j corresponds to the five size ranges, and $A_{j}$ is the virus size distribution based on the N-Amplicon levels obtained from one-step RT-qPCR analysis on the size-segregated cascade-impactor filters (i.e., Fig. 1A) at zero exposure time.

$F(t_{exp})$ represents the total percentage of aerosols remaining suspended at a given exposure time across all the size ranges. For example, after 5 mins, 93.03 ± 13.23% of the PRCV aerosols remained suspended in the air, at 65–75% RH levels. After 10, 20, 30, and 40 min, 87.38 ± 12.78%, 78.68 ± 12.05%, 72.08 ± 11.48%, and 66.74 ± 11.05% of the PRCV aerosols remained suspended, while only 58.29 ± 10.43% of PRCV particles remained suspended in the air after 60 min. A detailed description of the propagation of error analyses in evaluating $F(t_{exp})$ is provided in section VII of the supplemental material.

Viral particle concentrations in the aerosols were measured by quantifying the N gene. Deposited viral particles could not be collected. The viral concentration measured after experiments was affected by both deposition and RH inactivation. Though viral deposition happened, we could exclude the effects of deposition by applying the correction factor $F(t_{exp}$). Considering not all viral particles could be collected because of deposition, the removal ratio of the PRCV genome by RH inactivation was corrected from ${log}_{10}(C/C_{0})$ to $\log_{10}\left[C/\left(C_{0}\times F\left(t_{exp}\right)\right)\right]$, which equaled to ${log}_{10}(C/C_{0})$–$\log_{10}F(t_{exp})$.

To ensure that our results reflect the true stability of the virus, we have considered RH-dependent deposition effects. However, recovery efficiency remains a key consideration in aerosol studies and can affect overall quantification and interpretation. Recovery efficiency of aerosol collection devices varies significantly depending on the device and particle size. For instance, studies report for 5 mL SKC biosampler, the sampling efficiency for viable influenza is 0.5–5% (23); the collection efficiency of 20 mL SKC biosampler for viable influenza H1N1 is 5.6 ± 3.0% of the bioSampler (24). The sampling efficiency is highly related to particle distribution (25); for viruses that have a diameter between 30 and 100 nm,the collection efficiency is lower than 10%. The collection efficiency in our experiments based on one-step RT-qPCR quantification by using 20 mL SKC biosampler to collect aerosolized PRCV is around 2%, consistent with prior reports for influenza. To mitigate the instability of virus recovery, we maintained consistent experimental conditions (e.g., device type and flow rate) across all trials, varying only RH. Data were normalized to account for RH-dependent deposition rates, minimizing confounding effects of collection efficiency on viral degradation trends. For mechanistic comparisons, post-collection analyses were performed on the same sample, ensuring relative differences (e.g., treated vs untreated) were unaffected by absolute recovery rates. While low recovery efficiency is an inherent limitation of aerosol studies, our experimental design and normalization strategies ensure robust conclusions about RH-dependent viral stability.

### Exposure to RH compromised the integrity of the capsid and genome

**Fig 2:**
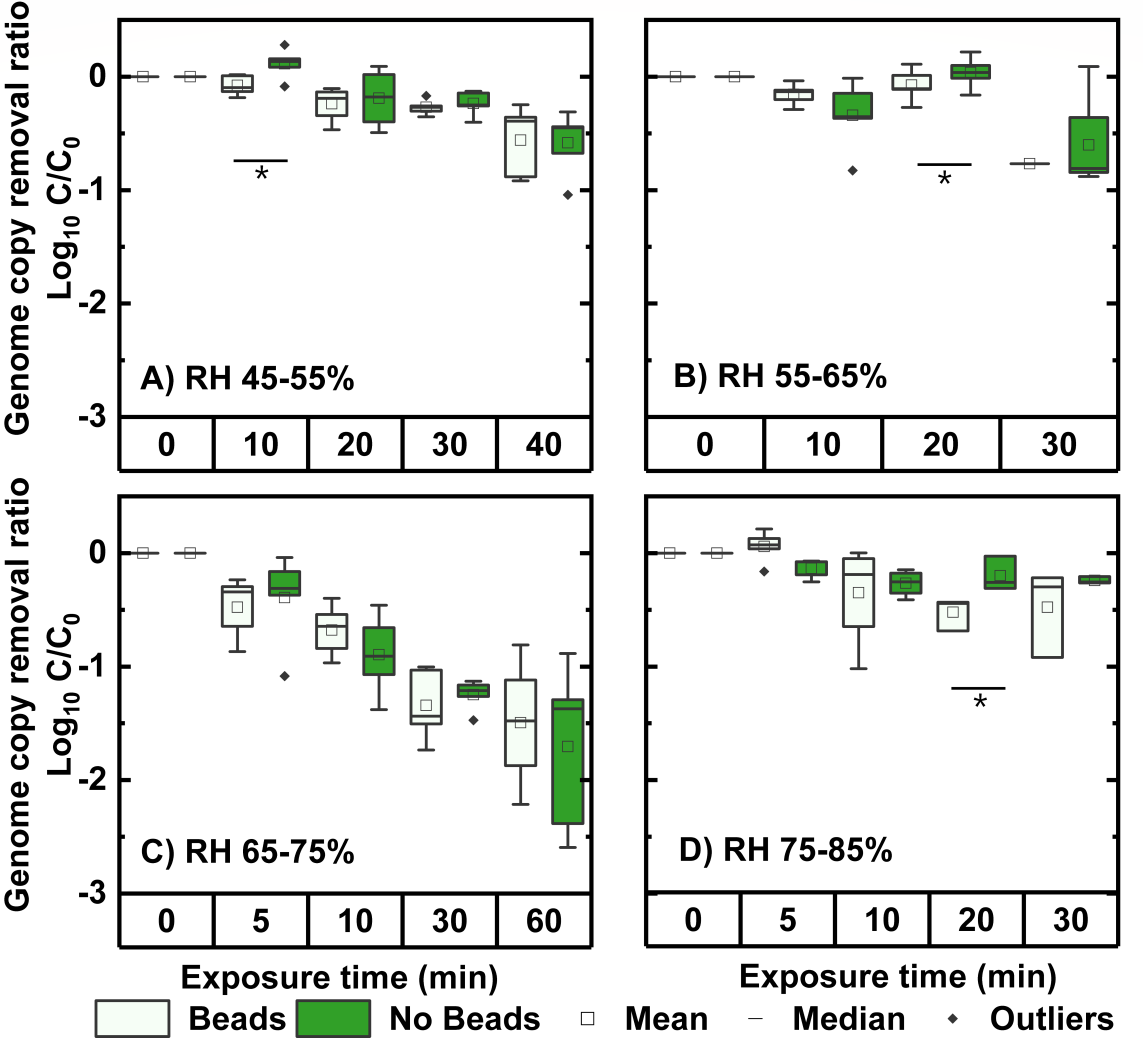
Effects of humidity on aerosolized PRCV binding to PGM-MB as measured by amplicon levels of the N gene. Five replicates were conducted for each time period at each RH level, and replicates above the LOQ were shown (at least three). Data were expressed as *C*/*C*_0_, where *C*_0_ was the average value of independent experiments from samples at 0 min for each RH condition. Data had been corrected from Log10 *C*/*C*_0_ to Log10 *C*/*C*_0_ – Log10 *F*($t_{exp}$) to exclude deposition effects. Data were expressed as box plots, showing means, medians, and outliers. In each RH level, at certain periods, beads-treated sample data (light green) were compared with no beads-treated sample data (dark green; i.e., control). Note that a significantly lower value for beads-treated samples as compared to untreated samples at a given time point indicates a damaged spike protein. Asterisks indicate significant differences (*P* < 0.05) between comparison groups. See the scatter plot in Fig. S8.

We aimed to identify how viruses are inactivated at different RH values on a mechanistic level. We first examined if RH treatment prevented PRCV from binding to its receptor by using a previously developed assay that quantifies PRCV binding to receptors found in porcine gastric mucin (PGM) by loading PGM onto magnetic beads (MBs) (26, 27). If the coronavirus Spike (S) protein, the protein that triggers attachment to the host cells, is damaged by RH, then virus binding to PMG-MBs would decrease. This decrease in viral particles with intact S protein can be quantified by a decrease in the PCR-based amplification of the PRCV nucleocapsid (N) gene after PGM-MBs binding (28). Figure 2 compares N gene amplicon levels from aerosolized viruses that were incubated in the presence or absence of PGM-MBs after the aerosolized viruses were exposed to different RH levels at different times. Figure 2 shows that RH did not alter virus binding to PGM-MB at any of the RH values tested: the values for N were similar either in the presence or absence of PGM-MBs. Indeed, among the 15 tested conditions, the data from RH-treated viruses with PGM-MB showed significantly smaller values compared to those without PGM-MB in three cases (*P* < 0.05, paired *t*-test). These include RH 45–55% for 10 min, RH 55–65% for 20 min, and RH 75–85% for 20 min. At these RH levels, we did not observe the trend that RH had an apparent impact on spike protein because a single time point was not supportive.

The next question was if RH affected capsid integrity. To answer this question, the same aerosolized virus samples were treated with RNase A/T1. If N proteins oligomerize to protect the viral genome, then the genome should be resistant to RNase A/T1. If the genome is not protected by the capsid, then RNase A/T1 will degrade it (29). In this case, we used a long-range RT-qPCR assay to determine if RH decreases capsid integrity. This assay consisted of three steps. In the first step, the N RNA, if present, was reverse transcribed into cDNA. In the second step, a long-range qPCR targeting N cDNA was used for amplification. In the third step, the N DNA (if present) was amplified again and quantified using qPCR. If the capsid integrity was disrupted, then exposed viral RNA would be degraded by RNase A/T1, preventing the amplification of the N gene.

**Fig 3:**
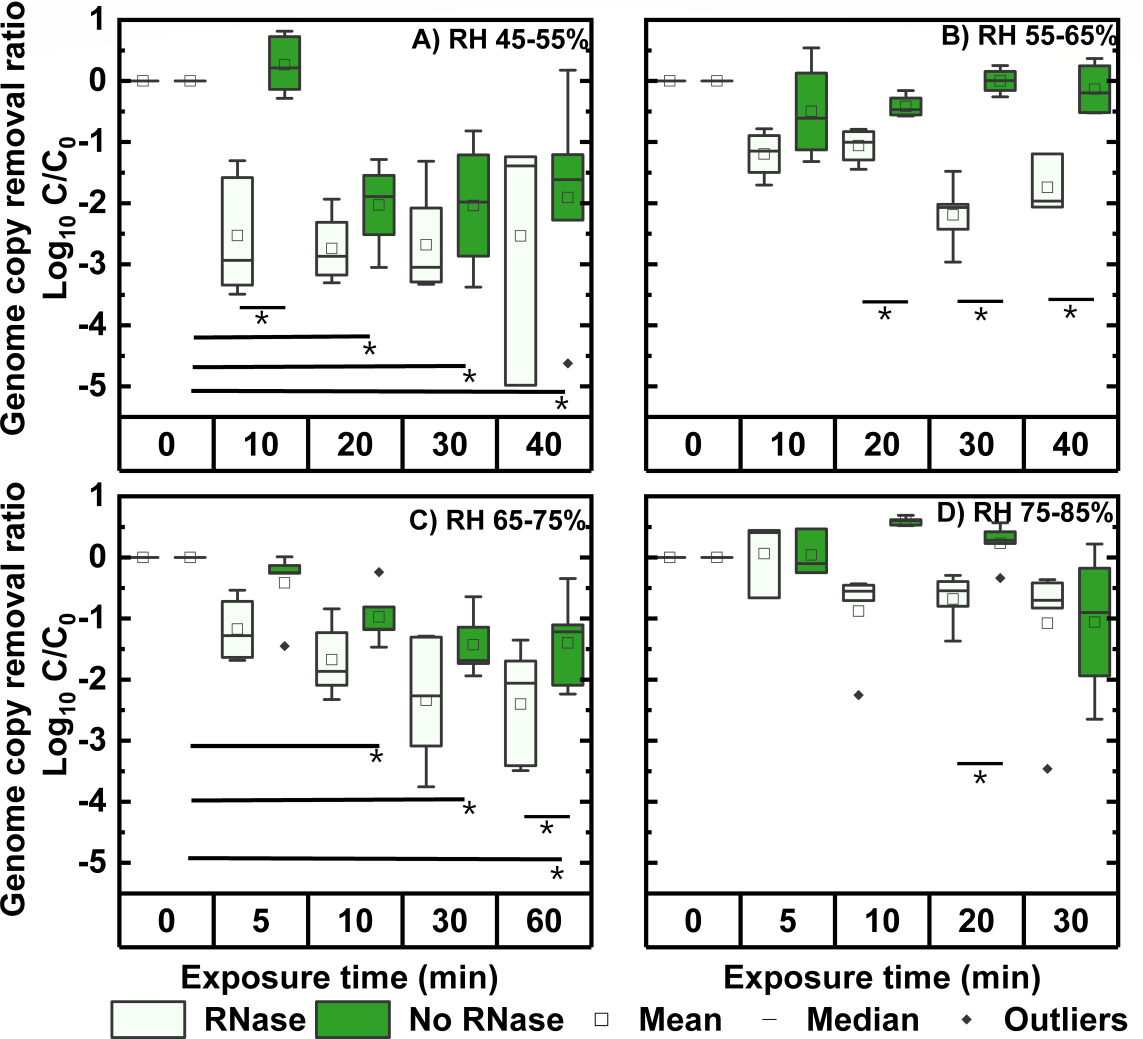
Effects of humidity on capsid stability and genome stability of aerosolized PRCV. Five replicates were conducted for each time period at each RH level, and replicates above the LOQ were shown (at least three). Data were expressed as *C*/*C*_0_, where *C*_0_ was the average value of independent experiments from samples at 0 min for each RH condition. Data had been corrected from Log_10_ *C*/*C*_0_ to Log_10_ *C*/*C*_0_ – Log_10_ $F(t_{exp})$ to exclude deposition effects. RNase-treated sample data (light green) were compared with no RNase-treated sample data (dark green). Note that a significantly lower value for RNase-treated samples as compared to untreated at a given time point indicates a damaged capsid. In addition, the untreated data (dark green) from other time points were compared with their value at 0 min for different RH conditions. A significantly lower value of an untreated sample at a given time point when compared to 0 min indicates genome damage. Asterisks indicate significant differences (*P* < 0.05) between comparison groups discussed in the text. See the scatter plot in Fig. S9.

Figure 3 shows the removal ratio of N gene amplicon levels from untreated versus RNase A/T1-treated samples. Significant reductions (*P* = 0.03, 0.04, and 0.017 < 0.05) from treating samples with RNase A/TI were observed for three time points at RH 55–65% (20, 30, and 40 min), and one time point each at RH 45–55% (10 min), RH 65–75% (60 min), and RH 75–85% (20 min) by conducting either paired *t*-test or Wilcoxon signed-rank test depending on data distribution. These findings suggest that RH exposure compromises capsid integrity, with the most consistent effects observed at RH 55–65%. The sporadic significance at other RH levels may reflect variability in the assay or sample processing.

Importantly, these results demonstrate how specific RH conditions can damage viral capsids, providing insights that could help predict the removal patterns of coronaviruses in certain indoor conditions.

Next, we evaluated whether RH could damage the viral genome itself by conducting the long-range RT-qPCR assay. In these assays, we assume that if a viral genome is degraded by RH, then it cannot be PCR amplified. Ideally, the whole viral genome should be examined for this assessment. However, the total PRCV genome is 27,550 bp long, making this technically challenging. Instead, we attempted to qPCR amplify a 1,562 bp fragment of the N gene, which is 5.6% of the PRCV genome. We quantified this genome fragment from PRCV-containing aerosols after exposure to various humidity levels for different durations. These aerosol samples did not undergo RNase treatment. The results of the long-range RT-qPCR of these samples are shown in Fig. 3. When examining the four time points across four different RH conditions, there were statistically significant differences (*P* < 0.05, paired *t*-test) between PRCV aerosol exposed in 0 min and other periods for RH 45–55% (20, 30, and 40 min), indicating that RH treatment resulted in RNA genome damage (Fig. 3A, no RNase). We also observed a trend in decreased N gene amplicon levels as exposure time increased at RH 65–75% (Fig. 3C, No RNase), and differences between the values at 0 min and those at 10, 30, and 60 min were statistically significant (*P* < 0.05, paired *t*-test). However, there was no significant reduction in N gene amplicon when aerosolized viruses were exposed to either RH 55–65% (Fig. 3B, No RNase) or RH 75–85% (Fig. 3D, No RNase). In summary, these results revealed that the PRCV genome was damaged when viral aerosols were exposed to a typical indoor environment, and the severity of this damage depended on RH.

Based on the data presented above, we conclude that exposure to inactivated PRCV at different RH values compromises the integrity of the viral genomes and capsids. However, as measured by the viral genome and capsid integrity, we did not observe a monotonic or V-shape trend in the relationships between RH level and inactivation mechanisms, but this could be due to the testing range being limited to 45-85%.

### Long-range RT-qPCR after RNase treatment reflects levels of PRCV infectivity loss

**Fig 4:**
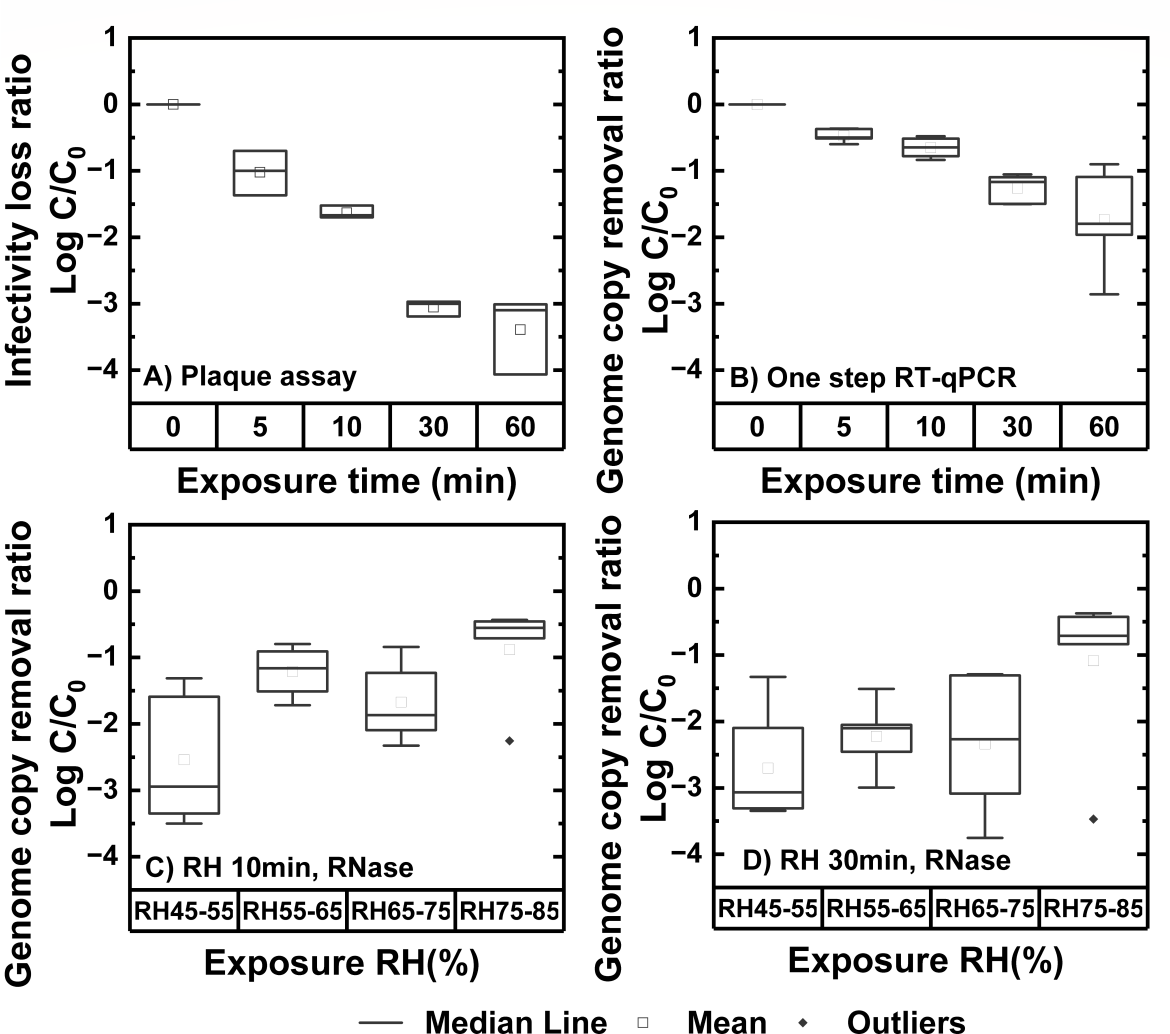
Effects of RH 65–75% on aerosolized PRCV as measured by (**A**) viral infectivity and plaque forming assays (conducted using $2\times 10^{6}$ PFU of PRCV once and $2\times 10^{7}$PFU twice), (**B**) N gene amplicon levels via one-step RT-qPCR (five replicates). Effects of various humidities on aerosolized PRCV as measured by N gene amplicon levels via long-range RT-qPCR with RNase A/T1 treatment at (**C**) 10 min and (**D**) 30 min RH exposure times (five replicates). Data were expressed as *C*/*C*_0_, where *C*_0_ was the average value of independent experiments from samples at 0 min at corresponding RH levels. Data had been corrected from Log_10_ *C*/*C*_0_ to Log_10_ *C*/*C*_0_ – Log_10_ *F*($t_{exp}$) to exclude deposition effects. Data were expressed as box plots. See the scatter plot in Fig. S10.

We examined virus infectivity via (i) plaque assays and (ii) N gene amplification reduction via different molecular assays. As shown in Fig. 4A, there was a decrease of 3 log_10_ in PFU over 30 min. On the other hand, as shown in Fig. 4B, for samples’ RNA measured by one-step RT-qPCR, only 1 log_10_ decrease in N gene amplicon concentration was observed over 30 min. We also conducted a paired *t*-test and found significant differences in three out of four comparisons (see section VI in the supplemental material for the *P* value). Based on these results, we concluded there is a more rapid decrease in PFU than N gene amplicon copies by at least an order of magnitude. As shown in Fig. 3C for samples without RNase treatment, only 1 log _10_ reduction in N gene amplicon concentration was observed over 30 min and the result of the paired *t*-test revealed insignificant differences between the results measured by one-step RT-qPCR and long-range qPCR, indicating the genome damage measured from both assays was at the same level for this RH (65–75%). However, the paired *t*-test (*P* = 0.05) between plaque assay data (Fig. 4A) and RNase assay data (Fig. 3C, RNase) for samples exposed to RH 65–75% indicated a significant difference (*P* = 0.012 for 1-h exposure) in only one out of four comparisons, which made RNase treatment with long-range RT-qPCR a better method to reflect infectivity loss of aerosolized virus.

Based on the conclusion above, we examined the viral genome removal ratio measured by N gene amplicon reduction via long-range RT-qPCR after RNase treatment across all RH levels at time 10 min (Fig. 4C) and 30 min (Fig. 4D). Results showed the highest viral stability occurred at RH 75–85%. With exposure time increased from 10 to 30 min, more genome removal occurred at RH 55–65% and 65–75%. A 3-log genome removal ratio was achieved at 10 min and did not increase with time anymore for RH 45–55%. Other time-based comparisons were conducted and are shown in Fig. S6 in section VII of the supplemental material.

## DISCUSSION

Complicated relationships existed between RH levels and the inactivation of aerosolized virus. In our study, PRCV exhibited the highest viability at the highest RH levels tested, but no monotonic relationship was found. A similar finding was reported previously for SARS-CoV-2 aerosol (17). Higher MERS-CoV viability was found at lower RH (40% RH vs 70% RH) (20). These differences were related to the viral types, spraying medium, other environmental conditions, and virus aerosolization/collection process (13). Thus, we concentrated on the inactivation mechanisms by examining the PRCV genome and capsid stability across different RH conditions and exposure times. The general data trends show genome damage occurred at RH 45–55% and RH 65–75%, and a compromised capsid was observed at RH 55–65%. A significant reduction of genome copies as measured by long-range RT-qPCR was observed at three out of four time points at RH 45–55% and RH 65–75%, and significant differences at three out of four time points in samples with and without RNase treatment were found at RH 55–65%. At the RH 75–85%, no significant damage to the genome or capsids was observed. Our data suggest that the virus is most stable at high RH (75–85%). However, when RH decreases, two interpretations arise: (i) the capsid is damaged for RH from 45% to 75%, and at RH 55–65%, the genome remains intact, but at RH45–55% and 65–75% the genome is damaged; (ii) the capsid is damaged only at RH 55–65% while the genome is damaged only at RH45–55% and 65–75%. The result’s interpretation was limited by the RNase assay. To access capsid damage level, we conducted an assay involving RNase treatment for the collected aerosols and compared the PCR amplicon concentrations for samples with and without RNase treatment. However, this comparison can determine whether the capsid is damaged only when the genome inside the virions is intact. If both capsid and genome have been compromised before RNase treatment, then the RNase assay cannot differentiate the capsid damage from another situation where only genome damage occurs. Although we did not measure the difference between N amplicon amplification by the RNase assay at RH 45–55% and 65–75%, we cannot rule out the possibility of both genome and capsid damage after exposure to these two RH conditions. Thus, while this method provides insight into capsid integrity, its interpretation is limited by the potential for simultaneous genome damage, highlighting the need for complementary approaches to distinguish these effects.

Multiple chemical mechanisms have been proposed to explain the inactivation of aerosolized viruses, including phase separation (30), crystallization (31), and shifts in pH (32). Based on our findings, we propose the following mechanism to explain RH-dependent viral inactivation: Under a high RH of 75–85%, aerosolized virions retain a higher hydration state. In contrast, at lower RH levels, accelerated dehydration may induce physicochemical alterations at the virion surface, such as solute accumulation from rapid water loss, potentially compromising its integrity and exposing the viral genome to environmental degradation. The observed correlation between reduced viral stability and lower RH aligns with this hypothesis. However, direct evidence of solute dynamics is beyond the scope of this study and needs further investigation. Previous studies have also related inactivation with either capsid damage (norovirus) (33) or genome damage (poliovirus) (34) or consider both (foot-and-mouth disease virus) (35). Thus, the findings here that RH may trigger inactivation via more than one mechanism are not without precedent.

Classical cell-based assays like plaque assay or end-point dilution assay (TCID_50_) identify the presence of infectious virus particles (36). These assays are expensive, labor/time-intensive, and not feasible for many viruses of public health relevance. Thus, researchers will instead quantify portions of a viral genome (e.g., using PCR-based methodology) as a proxy for infectious virus particles. Plaque assays for aerosolized viruses were examined for one RH condition (RH 65–75%) here, and the plaque assay data were compared with the other molecular assays performed at this same RH value. There was a strong correlative relationship (Pearson’s correlation *r* = 0.97) between an increase in the viral genome damage (as measured with RNase-treated samples) and a decrease in virus infectivity (as measured by plaque assay). However, it appears that the RT-qPCR assays (either the one-step or long-range assays) may overestimate the transmission risk for aerosolized virus particles; the plaque assays have much lower values than the PCR assays. Thus, going forward, when measuring the inactivation of aerosolized viruses, RNase treatment followed by long-range RT-qPCR may be the more accurate method to determine the presence of infectious virus particles.

There were several limitations in our study. First, we calculated the particle deposition in different size ranges, but the cascade impactor did not provide detailed size information for the smallest and largest particles collected. Second, the deposition coefficients measured using the OPS + SMPS data were for aerosolized PBS particles, due to safety and disinfection issues with using live viruses with these instruments. We acknowledge that this could introduce some bias if the deposition coefficient of aerosolized virus particles (from virus suspension in phosphate-buffered saline [PBS]) differs substantially from that of aerosolized PBS. Third, we used PBS as the viral stock dilution medium for aerosolization and collection rather than human fluid. PBS is widely used as the collection medium to ensure higher viability (37), and also widely used to dilute virus stock in aerosolization experiments (38, 39). However, PBS containing 0.9% salts and no proteins differs from that of human respiratory fluids. Consequently, the findings of this study, which utilized PBS as an aerosolization and collection medium, may not fully replicate results obtained using human fluids. Fourth, the particle size distribution was measured by N gene amplicon levels, which comprised both viable and non-viable vial aerosols. However, the plaque assay was selective for infectious viruses only. The correction factor *F* ($t_{exp})$ calculated with the size-distribution data cannot be applied to plaque assay data directly. Fifth, PGM-MBs can capture virions with some but not all intact spike proteins, and the detection provided by the PGM-MB assay can include virions with a certain number of compromised spike proteins. Sixth, the long-range qPCR reactions amplified part of the N region of the viral genome. As technology improves over time, it may be possible to get a more complete sequencing of the genome, including the ends of the genome. It would be interesting to see if genome stability or mutations are uniform or heterogeneous across the genome in the conditions tested here. Such results may allow us to better refine our understanding of how RH affects virus stability. Finally, as previously noted (40, 41), our system mimics indoor RH conditions within a range of 45–85%, which is constrained by the capabilities of our chamber system and the local humidities. It is important to acknowledge that lower indoor RH levels are common in many regions (42), which could potentially perform differently in the inactivation mechanisms of aerosolized PRCV.

Despite these limitations, the findings of this study provide valuable insights in identifying the biological mechanisms of PRCV viral aerosol inactivation for various RHs. The observation that RH effects are virus-specific underscores the need for pathogen-targeted engineering controls. However, the mechanistic framework we proposed and the trend between RH and virus inactivation we found can be generalized to evaluate the behavior of the coronavirus family in indoor conditions. By revealing that lower RH levels accelerate viral inactivation through dehydration-induced capsid damage, our work specifies RH levels with apparent virus activation. This provides a scientific basis for prioritizing humidity control in indoor spaces to reduce viral persistence. For example, maintaining RH levels within ranges that maximize viral inactivation (while balancing human comfort and energy efficiency) could become a standard engineering control to mitigate airborne transmission. Further studies are needed to (i) assess the impact of accumulated salts and fluctuations in pH on the integrity of capsids and their viral genomes (26), (ii) examine the effects of RH on viruses suspended in synthetic saliva or other synthetic body fluids, and (3) examine the viral inactivation mechanisms at low RH levels. While recent findings in aerosol chemistry may potentially play a role in virus decay, the specific mechanism of inactivation remains elusive (18, 28). Such studies are essential for a more comprehensive understanding of the mechanisms behind the RH-related effects by combining chemical and biological explanations and delving into a deeper understanding

## MATERIALS AND METHODS

### Virus preparation and cell culture

The swine testicular (ST) cell line (ST-CRL-1746; ATCC) was used for PRCV propagation. The PRCV ISU-1 (referred to as PRCV; Genbank DQ811787.1) sequence was verified by comparing the sequences of the nucleoprotein (N) gene region of the viruses grown in the lab with the sequences in the gene bank. The infectivity of PRCV was determined by plaque assay. We seeded ST cells into six-well tissue culture plates for overnight incubation. The cells in each well were first washed with PBS and then seeded with serially diluted PRCV samples. As described in previous studies (43, 44) with modifications that 80% confluence cells were required before seeding PRCV; 1.5 h of incubation were applied to the plates before the plates were overlaid with agar solution; and these plates were refrigerated to solidify agar, then incubated at 37°C and in 5% CO_2_ for 4 days. Then, the gel was carefully removed by adding a 10% formaldehyde solution. Cellular monolayers were fixed and stained using 0.05% crystal violet in 10% ethanol. Plaques were counted visually in the range of 1–10^7^ PFU.

See section I of the supplemental material for a detailed description of cell cultivation, PRCV sequencing, PRCV propagation, media formulation, and plaque assay.

### PRCV as a model for coronaviruses: key reasons

PRCV was used because swine coronaviruses (including PRCV) have a similar genome organization, replication strategy, and viral protein expression with other human and animal coronaviruses (45). As a mutant of TGEV, PRCV was previously used as a surrogate of SARS-CoV-2 (46, 47) because they show sufficient similarities as to genome length and virion structure (48). Besides SARS-CoV-2, PRCV is reported to have common histological characteristics to SARS-CoV infection (49). Also, PRCV is used as a surrogate of human CoVs in seawater (50).

### Aerosolization chamber

Virus aerosolization experiments were performed in a transparent plastic chamber with a volume (*V*) of around 44 L, placed inside a Class II biosafety cabinet. The AER of the chamber was 0.016 h^−1^ (measured using the CO_2_ decay method [51, 52], when all the vents were sealed and the nebulizer was turned off), indicating good sealing with minimal leaks (see Fig. S2 and its description in section II of the supplemental material for AER calculations). The chamber had four vent openings (nebulizer air inlet, aerosol sampling outlet, an extra inlet and outlet for the makeup or balance air), and a sealable door (see Fig. S3a in section III of the supplemental material for the chamber schematic). We placed an RH probe (ACU.RITE) inside the chamber to monitor the real-time RH.

Compressed air was filtered (Pall HEPA Capsule filter 12144) and pushed into the chamber balance air inlet to maintain the chamber pressure. This balanced air consisted of one dry and one humidified air stream. The flow rate of the dry and humidified air streams was regulated using flow control valves (Whitey SS-2RS4 Stainless Steel Valve 1/4) to achieve the desired RH in the chamber at the beginning of each experiment. Air was drawn from the balance air outlet using a vacuum pump and first passed through a HEPA capsule filter and a disinfection tank (filled with 10% bleach) before finally exiting the pump. We used another vacuum pump to draw and sample air from the chamber outlet. Sampled air passed through a bio-sampler (SKC, 20 mL, 225-9595), followed by a HEPA capsule filter and a disinfection tank. Fig. S3b depicts the sampling schematic for these experiments.

### Virus aerosolization

A nebulizer (Philips Respironics) was filled with 10 mL of diluted PRCV solution (1 mL virus stock and 9 mL PBS) and placed inside the chamber to generate aerosols. 2 × 10^6^ PFU (in 2 mL) PRCV was used for each aerosolization experiment. The virus stock contained 1× MEM solution and 1× antibiotic-antimycotic (refer to section I and Fig. S1 of the supplemental material for a description of media composition). Each experiment consisted of four steps (see Fig. S3b in section III of the supplemental material). Step 1, Chamber conditioning and setting of RH level: we opened the valves and turned on the airflow in the balance air inlet and outlet (12.5 LPM each), to make the chamber RH 15–20% below the desired chamber RH (45–55%, 55–65%, 65–75%, or 75–85%). So, in the following nebulization step, RH increase from aerosol generation will lead the final chamber RH to be located in the desired RH level. Step 2, Virus aerosolization: once the chamber reached the desired RH (typically 20% below the target RH), we turned on the nebulizer (6.5 LPM) for 5 min. The balanced air outlet was regulated at 6.5 LPM while sealing both the aerosol sampling outlet and air inlet. Step 3, Virus exposure: for all experiments here, the time point of 0 ($t_{exp}$ = 0）occurs when the nebulizer is turned off. The viruses were exposed in the chamber for different exposure durations ($t_{exp}$), as indicated by “exposure time” in the applicable figures. There were slight differences in the exposure times between experiments because we optimized each RH condition for the detection of the nucleocapsid (N) gene for one-step RT-qPCR. Specifically, we exposed PRCV aerosol for at most 60 min at RH 65–75% firstly and realized this exposure duration (i.e., $t_{exp}=60mins$) to be too long, without having a significant differentiation of virus genome removal compared to 30 minexposure duration. Thus, when we exposed PRCV aerosol in RH 75–85%, we shortened the longest exposure duration to 40 min. Also, it was found that a 5-min exposure duration was too short to show a significant difference with 0 min. Thus we removed the time points of 5 min when experimenting under RH 45–55% and RH 55–65%. Step 4, Virus collection: after the indicated exposure time in different RH values, we sampled aerosols (12.5 LPM) and simultaneously adjusted the balance air inlet to the same flow rate. RH-treated aerosolized viruses were collected for 15 min to remove around 99% of the suspended aerosolized viruses from the chamber (as determined using mass balance calculations in section III of the supplemental material). The viruses were suspended in 20 mL PBS for subsequent analyses (see below).

All experiments were conducted at room temperature (22.5 ± 0.6°C). The chamber was disinfected with 10% bleach, followed by 70% ethanol and then water between replicated experiments. In addition, the chamber was exposed to overnight UV radiation for an additional disinfection step. After virus aerosolization and chamber disinfection procedures as described above, water was aerosolized in the chamber, and those aerosols were collected and probed for the presence of N gene levels by using one-step RT-qPCR and the presence of infectious viruses using plaque assays to validate the efficiency of chamber disinfection. After disinfection procedures exhibited undetectable N gene levels as measured by the one-step RT-qPCR and undetectable infectivity as measured by plaque assay (more information is provided in section II of the supplemental material).

### Size-segregated collection of the viral aerosols

We quantified the size distribution of the collected aerosols by slightly modifying the experimental scheme described above (see Fig. S3c in section III of the supplemental material). PRCV was collected onto quartz filters placed in a five-stage cascade impactor (SKC Sioutas cascade impactor 225-370) instead of a bio-sampler during these experiments. The sampling flow rate during these experiments was 9 LPM. The flow rates of the other three vents were adjusted accordingly to maintain the chamber at atmospheric pressure. The cascade impactor segregated aerosols into five different size ranges (>2.5, 1.0–2.5, 0.5–1.0, 0.25–0.5, and <0.25 µm). Immediately after sampling, quartz filters were removed and transferred into vials containing 5 mL of PBS. These vials were sonicated (Cole-Parmer 8892) for 1 h, before RNA extraction and one-step RT-qPCR as described below. The cascade impactor was also disinfected similarly to the bio-sampler before subsequent experiments.

### Particle counting and deposition protocol

The deposition coefficients of aerosolized PBS solution were quantified by altering the experimental scheme (see Fig. S3d in section III of the supplemental material). The sampling outlet was connected to an optical particle sizer (TSI OPS 3330; OPS) and a scanning mobility particle sizer (TSI CPC 3750 and TSI Electrostatic Classifier 3082; SMPS 3938) operating in tandem to measure the size-segregated particle number concentrations (PNCs) over a wide size range of 10–10^4^ nm. The nebulizer was filled with PBS to mimic the virus aerosolization experiments. We measured PNC during aerosolization (i.e., when the nebulizer was turned on) and then for an additional 120 min after turning off to determine the particle deposition coefficients. The particle concentrations ($P_{t}$) in the chamber at time *t* can be depicted using a mass-balance approach with the following first-order differential equation (52, 53):


(5) $$\begin{array}{c} \frac{dP_{t}}{dt}=\frac{PER}{V}-\lambda P_{t}\; \end{array}$$

where PER is the particle emission rate when the nebulizer is turned on, and λ is the total loss coefficient of the particles. Once the nebulizer is turned off (time t = 0; PER = 0), we can calculate particle concentrations at time $t(P_{t}),$ as a function of particle concentrations at the end of nebulization ($P_{0};$ i.e., t=0), using the following equations:


(6) $$\begin{array}{cc} \frac{dP_{t}}{dt}=-\lambda P_{t}\to \int_{0}^{t}\frac{dP_{t}}{P_{t}}=\;\int_{0}^{t}-\lambda .dt & \\ P_{t}=P_{0}e^{-\lambda t} \end{array}$$

The λ value can be calculated from the experimentally determined PNC time series data by using a first-order exponential decay regression fit. The λ term consists of a deposition loss coefficient, k (including gravitational settling, wall loss, and Brownian diffusion loss), and sampling flow loss coefficient. The sampling flow loss coefficient is the ratio of the sampling flow rate (Q = 2 LPM) and chamber volume (V = 44 L). The deposition loss coefficient can be calculated by subtracting the sampling flow loss coefficient from the total loss coefficient, λ, as follows:


(7) $$\begin{array}{c} k=\lambda -\frac{Q}{V}=\lambda -\frac{2}{44}=\lambda -0.045\; \end{array}$$

This process was repeated for each size bin from 10 to 10,000 nm to obtain the deposition loss coefficient (k) as a function of particle diameter ($D_{p}$).

### Incubation of aerosolized viruses with PGM-MBs to detect virus-receptor interactions

PGM-MBs have been used in virus capture assays (27, 54, 55). The preparation of beads was the same as described previously (see section IV of the supplemental material for PGM-MBs preparation and validation information) (56). Aerosolized PRCV was collected at the indicated RHs and times as described above and collected in 20 mL PBS as described above. A 200 µL aliquot of this virus-containing solution was then used for experiments. Briefly, 200 µL aliquots of PRCV-containing PBS was added to 780 µL PBS in new tubes. Then either 20 µL PGM-MB beads or PBS alone (as a negative control) was added to tubes. The mixtures were shaken at 200 rpm for 30 min at room temperature. For those tubes containing PGM-MBs, the beads were washed with 1 mL PBS three times as follows: tubes were vortexed for 5 s and then placed on a magnetic bead attractor for 1 min. Then, supernatants were removed, and beads were resuspended in 1 mL of fresh PBS. After the last PBS wash, the beads were suspended in 140 µL PBS and stored at −20°C until RNA extraction. The virus-containing samples that did not receive PGM-MBs were not subjected to the magnetic bead concentrator or washes.

RNA was extracted for each sample using the Viral RNA Mini-kit (Qiagen), following a modified manufacturer’s protocol: the AW2 buffer was added twice to improve RNA elution efficiency. Then, RNA was eluted into 20 µL nuclease-free water (ThermoFisher) rather than the AVE buffer provided in the kit. Next, 3 µL of RNA from each extraction was used for one-step RT-qPCR to amplify and quantify the N gene, using the steps described below. Data for samples that were treated with or without magnetic beads were compared to each other.

### RNase treatment to detect viral capsid stability

For some experiments, aerosolized PRCV was collected in 20 mL PBS and then incubated with either RNase A/T1 mix (ThermoFisher) or PBS. Either 500 µL or 200 µL (as a negative control) of the virus-containing solution was added to new tubes. A total of 50 µL of a 50-fold RNase A/T1 mix (ThermoFisher) and 50 µL of 50-fold SUPERase•In RNase Inhibitor (ThermoFisher) were added into the 500 µL aliquot following the manufacturer’s protocol (see section IV of the supplemental material for RNase treatment procedures and their validation). For the tube containing the 200 µL aliquot of the virus-containing solution, 100 µL nuclease-free water (Corning) was added. Next, RNA was extracted from each experimental reaction using the QIAamp Viral RNA Mini Kit (Qiagen) and was eluted into 20 µL nuclease-free water. RNA samples were then subjected to long-range RT-qPCR as described below. RNase can only react with viral RNA when the capsid has lost its integrity (57). Thus, if qPCR amplicon concentration decreases after viruses are treated with RNase, then this means that the capsid integrity is compromised.

### One-step RT-qPCR and long-range RT-qPCR to determine genome damage

Both one-step RT-qPCR and long-range RT-qPCR are used to quantify the N gene. One-step RT-qPCR is commonly used to quantify short gene pieces (58), in our case, 93 bp. Long-range RT-qPCR is used to amplify long gene pieces, thus providing a more accurate view of whether the whole genome is damaged compared to one-step RT-qPCR (59). Ideally, the whole viral genome should be examined. However, the total PRCV genome is 27,550 bp long, making this technically challenging. Instead, we developed a long-range RT-qPCR assay that amplified a 1,562 bp fragment of the N gene, which is 5.6% of the PRCV genome.

The SYBR-based one-step RT-qPCR reaction contained 3 µL of the sample with 5 µL 2× iTaq Universal SYBR Green 1-Step Reaction Mix (Bio-Rad Laboratories), 0.125 µL iScript Reverse Transcriptase (Bio-Rad Laboratories), 0.3 µL (10 uM) forward primer (5′-TCCTGGTGGTCTTTCAACCC-3′; IDT), 0.3 µL (10 uM) reverse primer (5′-CAGTTGGCACACCTTCGAGA-3′; IDT), and 1.275 µL nuclease-free water (Corning, NY, USA). The primers target the N gene. The 10 µL mixture was placed in one well of a 96-well plate (4306737, Applied Biosystems, USA) and reactions were run using a qPCR system (Quant Studio 3, Thermo Fisher Scientific, USA) with the following thermal cycle: 1 cycle (50°C for 10 min and 95°C for 1 min) and 40 cycles (95°C for 10 s, 60°C for 30 s). Long-range RT-qPCR assay consisted of three steps. In the first step, the N RNA was reverse-transcribed into cDNA: 6 µL of each RNA extract was reverse-transcribed using 2 µL PRCV-Reverse primer (5′-TACCACCTCTTGCTCTGACCT-3′; IDT), 10 µL M-MuLV Reaction Mix (New England BioLabs) and 2 µL M-MuLV Enzyme Mix (New England BioLabs). The mixtures were incubated at 42°C for 60 min, followed by 80°C for 5 min in MyCycler Thermal Cycler (Bio-Rad Laboratories). In the second step, a long-range PCR targeting N cDNA was used for amplification: reactions were placed on ice for a short period. A total of 3 µL of each reaction was incubated with 0.6 µL of 10 µM Forward primer (5′-TCCTGGTGGTCTTTCAACCC-3′; IDT), 0.6 µL of 10 µM Reverse primer (5′-TACCACCTCTTGCTCTGACCT-3′; IDT), 10 µL SYBR-green supermix (Bio-Rad Laboratories), and 5.8 µL nuclease-free water (Corning, NY, USA). The thermal condition of long-range PCR for PRCV cDNA was as follows: 1 cycle of 95°C for 2 min, 25 cycles of 95°C for 15 s, 55°C for 30 s, and 72°C for 90 s using the Applied Biosystems QuantStudio 3 Real-Time PCR System. In the third step, the N DNA was amplified again and quantified using qPCR targeting 93 bp: DNA product from the long-range PCR step was diluted 10 times with nuclease-free water (Corning, NY, USA) before use. The qPCR reaction was initiated by mixing 2 µL of the diluted DNA, 7.5 µL of SYBR Green Master Mix (Bio-Rad Laboratories), 0.375 µL of 10 µM forward primer (5′-TCCTGGTGGTCTTTCAACCC-3′; IDT), 0.375 µL of 10 µM reverse primer (5′-CAGTTGGCACACCTTCGAGA-3′; IDT), and 4.75 µL nuclease-free water (Corning, NY, USA). The Applied Biosystems QuantStudio 3 Real-Time PCR System was used.

For the qPCR analysis, we conducted a melting curve analysis for each primer set. We ensured that, for every PCR reaction, both the positive control and the samples exhibited consistent melting temperatures, confirming the specificity of the amplification. The resulting CT values from both one-step RT-qPCR and long-range RT-qPCR were converted into corresponding genome copy concentrations by fitting the CT into a standard curve. The synthetic dsDNA (IDT) was serially diluted (10^1^ to 10^7^ copies/μL) and qPCR amplified at the same time and in the same plate containing extracted RNA samples. Data were expressed as the removal ratio, as Log_10_ *C*/*C*_0_, where *C*_0_ was the average value of five independent experiments from samples at 0 min time for each different RH condition, and *C* was the value of five independent experiments at other exposure periods for various RHs. More details are described in section V of the supplemental material, following MIQE guidelines (60).

### Statistical method

Data normality was checked by conducting the Shapiro-Wilk test at *α* = 0.05. Paired data were analyzed by Paired *t*-test (for normally distributed data) or Wilcoxon signed-rank test (for non-normally distributed data) at *α* = 0.05. The following comparisons were conducted: bead-treated versus no-bead-treated, RNase-treated versus no RNase-treated, long-range RT-qPCR data at different periods for no RNase-treated samples vs. data obtained before exposure (at 0 min) and one-step RT-qPCR data versus plaque assay data, bead-treated data, and RNase-treated data. A comparison between bead-treated data and no bead-treated data reflected damage to the spiking protein, while a comparison between RNase-treated data and no RNase-treated data gives information on the damage of PRCV capsid. The comparison between bead-treated data and RNase-treated data with one-step RT-qPCR reflected whether the one-step RT-qPCR method could be used to estimate viral infectivity reduction led by damage on spiking protein and capsid. Comparison between plaque assay data and one-step RT-qPCR reflected whether the one-step RT-qPCR method could be used to estimate viral infectivity loss. The values of mean, median, and outliers were calculated for each data set to make box plots. Outliers refer to sample data that exceed 1.5 times the interquartile range (the difference between the third quartile and the first quartile) from the box. Statistical analysis values are provided in section VI of the supplemental material. For each period at each humidity, five independent replicates were collected. The sample replicates were treated using different assays. Data below the limit of quantification (LOQ) of the qPCR assay were excluded from the analysis, provided that there were at least three replicates above the LOQ. If fewer than three replicates were above the LOQ for any given RH and time condition, another set of three independent replicates was collected. Coefficient of variance for *t* = 0 at different RH levels under each treatment assay is shown in Table S9.

## AFTER EPUB

*[This article was published on 7 April 2025 with a missing author affiliation. The affiliations were corrected in the current version, posted on 16 April 2025.]*

## SUPPLEMENTAL MATERIAL

Supplemental material aem.02319-24-s0001.pdf Figures S1 to S10; Tables S1 to S9.

MIQE information aem.02319-24-s0002.xlsx Details of essential and desired information required in MIQE guidelines for opportunistic pathogen detection.

ASM does not own the copyrights to Supplemental Material that may be linked to, or accessed through, an article. The authors have granted ASM a non-exclusive, world-wide license to publish the Supplemental Material files. Please contact the corresponding author directly for reuse.
