# Supplementary material for: Capsid and genome damage are the leading inactivation mechanisms of aerosolized porcine respiratory coronavirus at different relative humidities
Source: Appl Environ Microbiol. 2025 Apr 7;91(5):e02319-24. doi: 10.1128/aem.02319-24 (PMC12094281; doi:10.1128/aem.02319-24)
Supplement: Supplemental material — Figures S1 to S10; Tables S1 to S9. [file aem.02319-24-s0001.pdf]

SUPPLEMENTARY INFORMATION

# Capsid and genome damage are the leading inactivation mechanisms of aerosolized porcine respiratory coronavirus at different relative humidities

Aijia Zhou[a]#, P. S. Ganesh Subramanian[a], Salma El-Naggar[b], Joanna L. Shisler[c], Vishal Verma[a], Thanh H. Nguyen[a,d,e]

[a]Department of Civil and Environmental Engineering, University of Illinois at Urbana-Champaign

[b]Department of Bioengineering, University of Illinois at Urbana-Champaign

[c]Department of Microbiology, University of Illinois at Urbana-Champaign

[d]Institute of Genomic Biology, University of Illinois at Urbana-Champaign

[e]Carle Illinois College of Medicine

#Address correspondence to Aijia Zhou, zaijia2@illinois.edu.

This supporting information document contains the following information:

7 sections, 10 figures, 9 tables, 1 Excel file, 28 pages

Section I: Virus preparation and Cell culture

**Figure S1**: Formulation of the minimum essential medium (MEM).

Section II: Air Exchange rate, virus aerosolization and chamber disinfection

**Figure S2**: Logarithmic decay plot of $CO_2$ concentration over time in the chamber to calculate air exchange rate.

Section III: Experimental configuration and protocols.

**Figure S3**: Schematics and protocols for PRCV aerosolization, PRCV collection, and particle counting.

Section IV: PGM-MBs preparation and RNase treatment

**Table S1:** Linear fitting models of relationships between PRCV genome copies and infectivity under different treatments

**Figure S4**: Relationships between PRCV genome copies and infectivity under different. treatments

Section V: One-step RT-qPCR and long range RT-qPCR.

**Table S2.** PCR primers, sequences, and thermal conditions

**Table S3**: Comparison of data from PCR assays fitted assuming first-order decay model

**Table S4**: Comparison of data from PCR assays fitted assuming second-order decay model

Section VI: Data statistical analysis

Section VII: Propagation of error in calculating F

**Figure S5**: Effects of various humidities on PRCV particle distribution as measured by. N gene amplicon levels via one-step RT-qPCR after cascade impactor collection and sonication

**Tables S5-8**: F-values and their standard deviation at RH levels: 45-55%, 55-65%, 65-75%, and 75-85%

**Figure S6:** Effects of various humidities on spike protein as measured by N gene amplicon levels via one-step RT-qPCR at certain time periods

**Figure S7-S10:** Box and scatter plots of Figure 1-4 in the main text.

**Figure S7:** Particle distribution and deposition coefficient after nebulization

**Figure S8:** Effects of humidity on aerosolized PRCV binding to PGM-MB as measured by N gene levels

**Figure S9:** Effects of humidity on capsid stability of aerosolized PRCV

**Figure S10:** Effects of RH 65-75% on aerosolized PRCV as measured by (A) Viral plaque forming assays (B) N gene amplicon levels. Effects of various humidities on aerosolized PRCV as measured by N gene amplicon levels via long-range RT-qPCR with RNase A/T1 treatment at (C) 10 minutes and (D) 30 minutes RH exposure times.

**Table S9:** Coefficient of variance (CV) for t=0 at different RH levels under each treatment assay

Details of essential and desired information required in MIQE guidelines for opportunistic pathogen detection (**Supplementary Excel**)

**Section I: Virus preparation and Cell culture**

Cell culture: ST cells were grown in Minimum Essential Medium (MEM; Corning) supplemented with 10% fetal bovine serum (FBS; ThermoFisher) and 1X Antibiotic-Antimycotic (ThermoFisher). ST cells were incubated at 37 $^0$C and 5% $CO_2$. ST cells were subcultured until monolayers reached 80% confluence. Monolayers were washed with PBS (ThermoFisher) and then incubated with a 0.25% trypsin/EDTA (ThermoFisher) solution until cells detached from the tissue culture flasks. Next, ST cells were passaged in a 1:3 ratio to keep cells growing in an exponential growth phase. Figure S1 provides a detailed description of MEM formulation. 100 X Antibiotic- antimycotic solution contains 10,000 units/mL of penicillin, 10,000 µg/mL of streptomycin, and 25 µg/mL of Gibco Amphotericin B. Thus, 1X Antibiotic- antimycotic solution contains 100 units/mL of penicillin, 100 µg/mL of streptomycin, and 0.25 µg/mL of Gibco Amphotericin B, as per the manufacturer's description.[1]

PRCV sequencing: PRCV N gene region was first reversed transcribed into cDNA using the reverse primer described below. Then the cDNA was amplified by PCR (Forward:5'-TCCTGGTGGTCTTTCAACCC-3'; Reverse:5'-TACCACCTCTTGCTCTGACCT-3'). After PCR purification by ExoSAP-IT™ PCR Product Cleanup Reagent Kit (ThermoFisher), the purified PCR product was subjected to gel electrophoresis and Sanger sequencing.

PRCV propagation: Subconfluent ST cellular monolayers were washed twice with 3 mL of PBS to remove all traces of the cell culture medium. Cellular ST monolayers in 175 $cm^2$ flasks were incubated with 1 mL PRCV ($10^5$ PFU/mL) per flask. After a 1-hour incubation with gentle shaking every 15 minutes, monolayers were washed with PBS to remove unattached PRCV. Then, 8 mL MEM (lacking FBS but containing Antibiotic-Antimycotic solution) was added to each flask. When at least 80% of the cellular monolayer was detached (usually three days), cells and supernatants were collected and centrifuged at 2,000 rpm for 5 minutes. Virus-containing supernatants were collected, aliquoted into 1.5 mL microcentrifuge tubes, and then stored at -80 $^0$C. Virus titers were determined by plaque assays, as described below.

Plaque assay: After achieving at least 80% confluence, ST cells in each well were first washed with 0.5 mL PBS. Viruses were serially diluted into MEM lacking FBS. Cellular monolayers were incubated with 400 uL of each serially diluted sample for 1.5 hours with shaking every 15 minutes. Media were then removed, and the plates were overlaid with 1% agarose–MEM in the presence of 2X MEM, 7.5% sodium bicarbonate, Antibiotic-Antimycotic solution, and 15 mM HEPES. These plates were refrigerated for 20 min until the agar solidified, then incubated at 37 °C and in 5% $CO_2$ for four days. Then, the gel was carefully removed by adding a 10% formaldehyde solution (in PBS) into each well for 2 hours. Cellular monolayers were fixed and stained using a solution containing 0.05% crystal violet in 10% ethanol.

## Minimum Essential Medium (MEM)
### Formulation

| Cat. No. | 50-004 | 50-010 | 50-019 | 50-011 | 51-010 | 90-009 | 10-009 | 10-010 | 15-010 | 15-015 | 17-305 |
|---|---|---|---|---|---|---|---|---|---|---|---|
| Description | Powder | Powder | Powder | Powder | Powder | Powder | Liquid, 1x | Liquid, 1x | Liquid, 1x | Liquid, 1x | Liquid, 1x |
| Units | mg/L | mg/L | mg/L | mg/L | mg/L | mg/L | mg/L | mg/L | mg/L | mg/L | mg/L |
| **Components** | | | | | | | | | | | |
| *Inorganic Salts* | | | | | | | | | | | |
| $CaCl_2$ (anhydrous) | 200.00 | 200.00 | 140.00 | 200.00 | 200.00 | 200.00 | 200.00 | 200.00 | 200.00 | -- | 200.00 |
| $Fe(NO_3)_3 \cdot 9H_2O$ | 0.10 | -- | -- | -- | -- | -- | -- | -- | -- | -- | -- |
| KCl | 400.00 | 400.00 | 400.00 | 400.00 | 400.00 | 400.00 | 400.00 | 400.00 | 400.00 | 400.00 | 400.00 |
| $KH_2PO_4$ | -- | -- | 60.00 | -- | -- | -- | -- | -- | -- | -- | -- |
| $MgSO_4$ (anhydrous) | 97.67 | 97.70 | 97.67 | 97.70 | 97.00 | 97.70 | 97.70 | 97.70 | 97.70 | -- | 97.70 |
| NaCl | 6400 | 6800 | 8000 | 6800 | 6800 | 6800 | 6800.00 | 6800.00 | 6800.00 | 6800.00 | 6800.00 |
| $NaH_2PO_4 \cdot H_2O$ | 124.00 | 140.00 | 47.88 | 140.00 | 140.00 | 140.00 | 140.00 | 140.00 | 140.00 | 1217.00 | 140.00 |
| Na succinate $\cdot 6H_2O$ | -- | -- | -- | -- | 100.00 | -- | -- | -- | -- | -- | -- |
| $NaHCO_3$ | -- | -- | -- | -- | -- | -- | 1500.00 | 2200.00 | 2200.00 | 2200.00 | 2200.00 |
| *Amino Acids* | | | | | | | | | | | |
| L-Alanine | -- | -- | | 8.90 | -- | -- | 8.90 | -- | -- | -- | -- |
| L-Arginine • HCl | 42.00 | 126.40 | 126.00 | 126.40 | 126.40 | 126.40 | 126.40 | 126.40 | 126.40 | 126.40 | 126.40 |
| L-Asparagine $\cdot H_2O$ | -- | -- | -- | 15.00 | -- | -- | 15.00 | -- | -- | -- | -- |
| L-Aspartic acid | -- | -- | -- | 13.30 | -- | -- | 13.30 | -- | -- | -- | -- |
| L-Cystine • 2HCl | 31.28 | 31.20 | 31.39 | 31.20 | 31.10 | 31.20 | 31.20 | 31.20 | 31.20 | 31.20 | 31.20 |
| L-Glutamic acid | -- | -- | -- | 14.70 | -- | -- | 14.70 | -- | -- | -- | -- |
| L-Glutamine | 292.00 | 292.00 | 292.00 | 292.00 | -- | -- | 292.00 | 292.00 | -- | -- | -- |
| Glycine | -- | -- | -- | 7.50 | -- | -- | 7.50 | -- | -- | -- | -- |
| L-Histidine • HCl $\cdot H_2O$ | 21.00 | 41.90 | 42.00 | 41.90 | 41.90 | 41.90 | 41.90 | 41.90 | 41.90 | 41.90 | 41.90 |
| L-Isoleucine | 52.40 | 52.50 | 52.00 | 52.50 | 52.50 | 52.50 | 52.50 | 52.50 | 52.50 | 52.50 | 52.50 |
| L-Leucine | 52.40 | 52.50 | 52.00 | 52.50 | 52.50 | 52.50 | 52.50 | 52.50 | 52.50 | 52.50 | 52.50 |
| L-Lysine • HCl | 73.10 | 72.50 | 72.50 | 72.50 | 73.06 | 72.50 | 72.50 | 72.50 | 72.50 | 72.50 | 72.50 |
| L-Methionine | 15.00 | 15.00 | 15.00 | 15.00 | 14.90 | 15.00 | 15.00 | 15.00 | 15.00 | 15.00 | 15.00 |
| L-Phenylalanine | 33.00 | 32.50 | 32.00 | 32.50 | 33.02 | 32.50 | 32.50 | 32.50 | 32.50 | 32.50 | 32.50 |
| L-Proline | -- | -- | -- | 11.50 | -- | -- | 11.50 | -- | -- | -- | -- |
| L-Serine | -- | -- | -- | 10.50 | -- | -- | 10.50 | -- | -- | -- | -- |
| L-Threonine | 47.60 | 47.60 | 48.00 | 47.60 | 47.64 | 47.60 | 47.60 | 47.60 | 47.60 | 47.60 | 47.60 |
| L-Tryptophan | 8.00 | 10.00 | 10.00 | 10.00 | 10.20 | 10.00 | 10.00 | 10.00 | 10.00 | 10.00 | 10.00 |
| L-Tyrosine • 2Na $\cdot 2H_2O$ | 52.19 | 51.90 | 51.90 | 51.90 | -- | 51.90 | 51.90 | 51.90 | 51.90 | 51.90 | 51.90 |
| L-Tyrosine, free base | -- | -- | -- | -- | 36.00 | -- | -- | -- | -- | -- | -- |
| L-Valine | 46.80 | 46.80 | 46.00 | 46.80 | 46.90 | 46.80 | 46.80 | 46.80 | 46.80 | 46.80 | 46.80 |
| *Vitamins* | | | | | | | | | | | |
| Choline bitartrate | -- | -- | -- | -- | 1.80 | -- | -- | -- | -- | -- | -- |
| D-Calcium pantothenate | 2.00 | 1.00 | 1.00 | 1.00 | 1.00 | 1.00 | 1.00 | 1.00 | 1.00 | 1.00 | 1.00 |
| Choline chloride | 2.00 | 1.00 | 1.00 | 1.00 | -- | 1.00 | 1.00 | 1.00 | 1.00 | 1.00 | 1.00 |
| Folic acid | 2.00 | 1.00 | 1.00 | 1.00 | 1.00 | 1.00 | 1.00 | 1.00 | 1.00 | 1.00 | 1.00 |
| *i*-Inositol | 3.60 | 2.00 | 2.00 | 2.00 | 2.00 | 2.00 | 2.00 | 2.00 | 2.00 | 2.00 | 2.00 |
| Nicotinamide | 2.00 | 1.00 | 1.00 | 1.00 | 1.00 | 1.00 | 1.00 | 1.00 | 1.00 | 1.00 | 1.00 |
| Pyridoxine • HCl | 2.00 | 1.00 | 1.00 | 1.00 | 1.00 | 1.00 | 1.00 | 1.00 | 1.00 | 1.00 | 1.00 |
| Riboflavin | 0.20 | 0.10 | 0.10 | 0.10 | 0.10 | 0.10 | 0.10 | 0.10 | 0.10 | 0.10 | 0.10 |
| Thiamine • HCl | 2.00 | 1.00 | 1.00 | 1.00 | 1.00 | 1.00 | 1.00 | 1.00 | 1.00 | 1.00 | 1.00 |
| *Other* | | | | | | | | | | | |
| D-Glucose | 4500 | 1000 | 1000 | 1000 | 1000 | 1000 | 1000 | 1000.00 | 1000.00 | 1000.00 | 1000.00 |
| Phenol red • Na | 15.00 | 10.00 | 10.00 | 10.00 | 6.00 | -- | 10.00 | 10.00 | 10.00 | 10.00 | -- |
| Succinic acid | -- | -- | -- | -- | 75.00 | -- | -- | -- | -- | -- | -- |
| Sodium Pyruvate | -- | -- | -- | -- | -- | -- | 110.00 | -- | -- | -- | -- |
| $NaHCO_3$ | | | | | | | | | | | |
| Powder (g/L) | 2.75 | 2.20 | -- | 2.20 | 2.20 | 2.20 | -- | -- | -- | -- | -- |
| 7.5% Solution (mL/L) | 33.40 | 26.70 | -- | 26.70 | 26.70 | 26.70 | -- | -- | -- | -- | -- |
| L-Glutamine | | | | | | | | | | | |
| Powder (mg/L) | -- | -- | -- | -- | 292.00 | 292.00 | -- | -- | -- | -- | -- |
| 200 mM Solution (mL/L) | -- | -- | -- | -- | 10.00 | 10.00 | -- | -- | -- | -- | -- |

Figure S1: Formulation of the minimum essential medium (MEM).

**Section II: Air exchange rate, virus aerosolization and chamber disinfection**

***Air Exchange Rate:***

The air exchange rate ($AER$) was evaluated experimentally by measuring $CO_2$ concentration decay in the chamber. All vents were connected to closed valves to ensure no leakage. A burning candle was placed inside the chamber till it naturally extinguished (~ 30 mins, due to consuming $O_2$ in the chamber) leading to a $CO_2$ concentration exceeding 5000 ppm. $CO_2$ decay was monitored using $CO_2$ sensors (IAQ-Max $CO_2$ sensor) for over 24 hours [a reduction from 5000 ppm (upper limit of instrument detection) was only apparent after 12 hours].

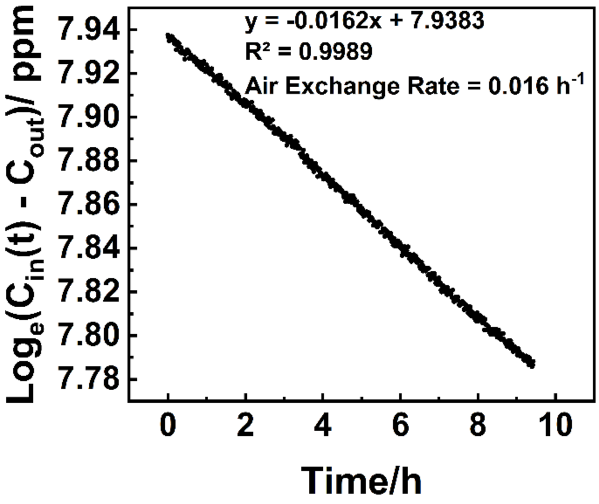


**Figure S2**: Logarithmic decay plot of $CO_2$ concentration over time in the chamber to calculate air exchange rate ($AER$).

In the absence of any source (i.e., after the candle extinguishes), $CO_2$ decay can be represented by the following mass balance equation:[2,3]

$$C_{in(t)} = (C_{in(0)} - C_{out})e^{-AER.t} + C_{out} \tag{1}$$

where $C_{in(t)}$, $C_{out}$, and $C_{in(0)}$ are the chamber concentrations of $CO_2$ at time $t$, steady state [i.e., ambient $CO_2$ concentrations outside the chamber (i.e., in the room where the chamber was situated; ~ 400 ppm)], and the initial concentration of $CO_2$ in the chamber (i.e., at beginning of decay; $t = 0$), respectively.

Equation 1 can be simplified to the following equation:

$$log_e\left(C_{in(t)} - C_{out}\right) = -AER.t + log_e\left(C_{in(0)} - C_{out}\right) \tag{2}$$

From equation 2, the slope of the $log_e\left(C_{in(t)} - C_{out}\right)$ vs. time can be used to determine the air exchange rate ($AER$). Figure S2 gives the logarithmic decay plot of $CO_2$ over time, from which the $AER$ was calculated to be 0.016 $h^{-1}$.

***Virus aerosolization:*** A nebulizer was placed inside the chamber through a sealable door. The nebulizer input tubing (flow rate = 6.5 LPM when the nebulizer was on) was then connected to the nebulizer before sealing the door. Note that all the chamber vents were sealed using a flow control valve before the start of each experiment. For the chamber RH level setting, the proportion of dry and wet air streams was adjusted accordingly to achieve the desired RH levels.

***Chamber disinfection***: by rinsing with 10% bleach (10 s rinsing followed by 30 s waiting) followed by 70% ethanol, and water (rinsing followed by 15 mins of soaking to prevent any residual disinfectant from influencing the subsequent experiment). The bio-sampler was then dried and cleaned using Kimwipe paper and filled with 20 mL of PBS for the subsequent experiments. The chamber remained shut until the samples obtained from the bio-sampler exhibited undetectable infectivity as measured by the plaque assay, which was at least overnight after exposure to UV radiation.

## Section III: Experimental Configuration and Protocols

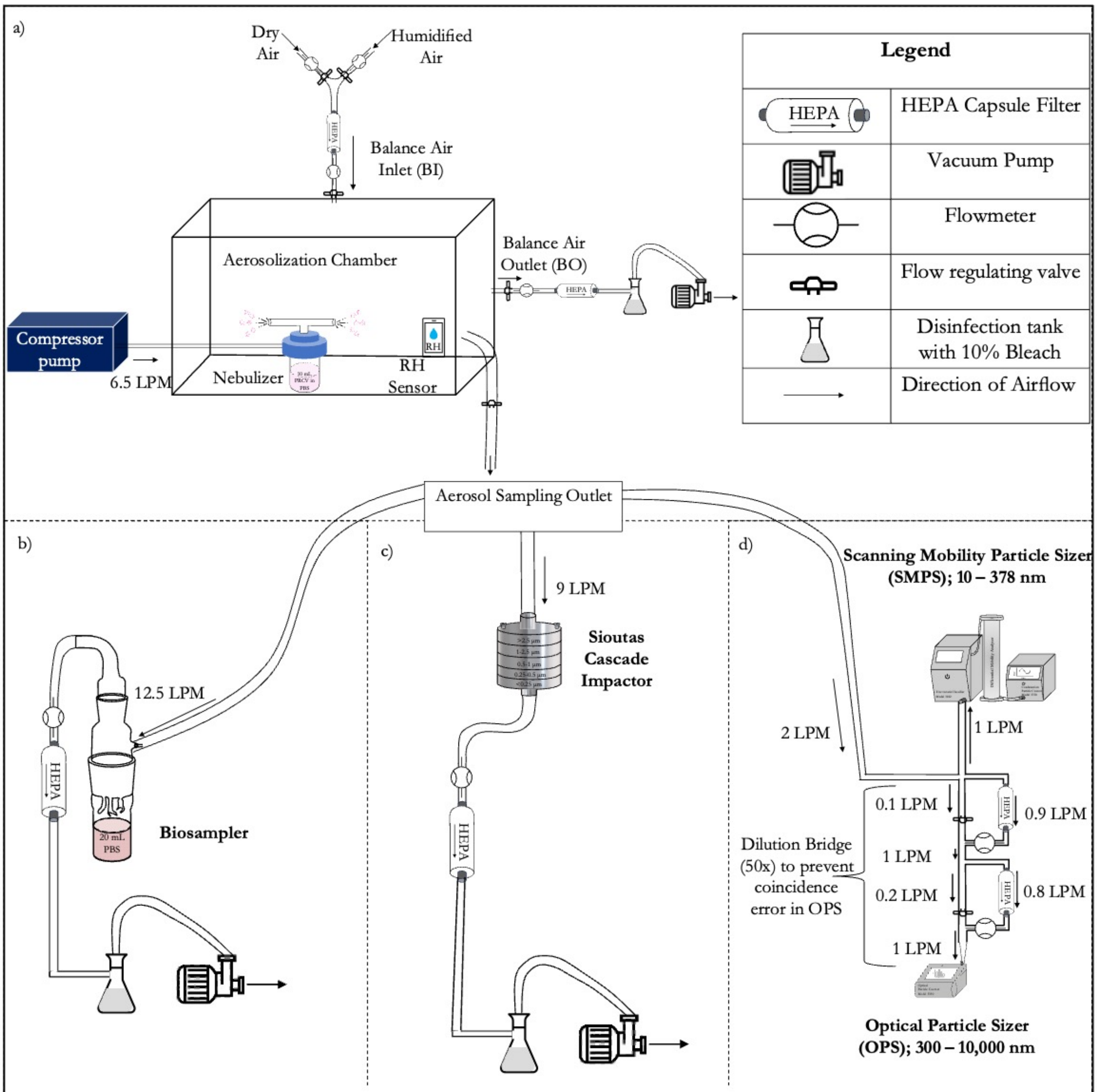


### Experimental Protocols

i.) ***Chamber Conditioning*** : Set BO and BI to 12.5 LPM flow rate to achieve desired chamber RH

ii.) ***Virus Aerosolization*** **:** Turn on the nebulizer (6.5 LPM), while turning off the BO and adjusting BI to 6.5 LPM for a total of 5 mins.

iii.) **Virus Sampling:** Turn off the Nebulizer. Turn on the sampling pump (12.5 LPM), and adjust the BI to 12.5 LPM for a total of 15 mins

i.) ***Chamber Conditioning*** : Set BO and BI to 12.5 LPM flow rate to achieve desired chamber RH.

ii.) ***Virus Aerosolization*** **:** Turn on the nebulizer (6.5 LPM), while turning off the BO and adjusting BI to 6.5 LPM for a total of 5 mins.

iii.) ***Virus Incubation:*** Close all the vents and turn off the nebulizer for a total of 0, 10, 20, or 30 mins.

iv.) **Virus Sampling:** Turn on the sampling pump (9 LPM), and adjust the BI to 9 LPM for a total of 15 mins

i.) ***PBS Aerosolization*** **:** Turn on the nebulizer (6.5 LPM), while adjusting the BO to 4.5 LPM, and start the sampling (2 LPM).

ii.) ***PBS Aerosol Decay*** **:** Turn off the Nebulizer and the BO, while turning on the BI to 2 LPM flow rate.

**Figure S3**: Experimental configuration and protocols for the a.) Aerosolization chamber, b.) Biosampler experiments, c) cascade impactor experiments, and d.) particle counting experiments.

***Aerosolized Virus Removal Percentage***

In the absence of any aerosol sources, the concentration of particles in the chamber at time, $t$ can be represented using the following mass balance equation:

$$P_t = P_0 \cdot e^{-\lambda \cdot t}, \quad (3)$$

where $P_0$ is the particle/aerosol concentration at $t = 0$ min (i.e., at the start of sampling), and $\lambda$ is the total loss coefficient of the particles [i.e., $\lambda = k + Q/V$ (sum of the deposition loss coefficient $k$, and flow losses $Q/V = flowrate/volume$)]. Equation 3 can be rearranged to obtain:

$$P_t = P_0 \cdot e^{-\frac{Q}{V} \cdot t} \cdot e^{-k \cdot t} \quad (4)$$

After 15 minutes of sampling the particle concentration in the chamber can be represented using:

$$P_{15} = P_0 \cdot e^{-\frac{12.5}{44} \cdot 15} \times e^{-k \cdot 15} = 0.014 \times P_0 \cdot e^{-k \cdot 15} \quad (5)$$

Since $e^{-k \cdot 15}$ is less than 1, $P_{15} < 0.014\, P_0$, indicating that at 15 mins sampling, around 99 % of the suspended aerosols were removed due to sampling and deposition losses.

**Section IV: PGM-MBs preparation and RNase treatment**

***PGM-MBs preparation***: MagnaBind carboxyl beads (ThermoFisher) were washed thrice with PBS. Ten mg of the mucin from porcine stomach (Millipore Sigma) was dissolved in 1 mL buffered ethanesulfonic acid (MES, ThermoFisher). 500 uL of 1-ethyl-3-(3-dimethylaminopropyl)carbodiimide hydrochloride) (EDC, ThermoFisher) was prepared as a cross-linking solution by dissolving 5 mg of EDC in 500 uL of MES conjugation buffer. 1 mL of the mucin solution was added to the washed beads, followed by an immediate addition of 0.1 mL of the EDC solution at room temperature. The beads were rotated gently at around 8 rpm for 30 minutes at room temperature, and the magnetic separation rack was used to separate the beads from the solution. The beads were washed three times using PBS, and the beads were resuspended in 1 mL of PBS with 0.05% sodium azide and stored at 4°C.

***Validation of PGM-MBs assay:*** 10^6 PFU/mL of PRCV was serially diluted till reaching 10 PFU/mL, thus, there were five different dilutions. For each dilution, we prepared 4*200uL of the PRCV samples. We conducted four experiments to 1. provide a dynamic range of PGM-MBs assay 2. validate that PGM-MBs could effectively capture virion only with completed spike protein.

Set 1: We did RNA extraction directly to 5 serial diluted PRCV samples using the Qiagen viral RNA mini kit

Set 2: 20uL of PGM-MBs were added into serial diluted PRCV samples as described in the PGM-MBs assay, after 30mins' shaking and PBS washing, we did RNA extraction to those PGM-MBs captured virus

Set 3: 2uL of Proteinase K(NEW ENGLAND Biolabs) was added into serial diluted PRCV samples to damage PRCV protein. After incubating the samples for 15 minutes at 37 Celsius degree, we heated the samples at 95 Celsius degree for 10 minutes to inactivate the Proteinase K. Then we did RNA extraction to those protein-damaged viruses.

Set 4: Similar to Set 3, Proteinase K was applied to serial diluted PRCV samples to cause protein damage, followed by heat inactivation. Then 20uL of PGM-MBs were added to these protein-damaged viruses. After 30 minutes of shaking and PBS washing, we did RNA extraction to check whether these protein-damaged viruses can be captured by PGM-MBs or not.

Then the extracted RNA was quantified using one-step RT-qPCR, measured by amplicon levels

Table S1: Linear fitting models of relationships between PRCV genome copies and infectivity under different treatments

| Set Order | Linear Fitting Model | R Square |
|---|---|---|
| Set 1 (RNA extraction) | y = 0.8785x + 2.6065 | 0.9891 |
| Set 2 (bead before RNA extraction) | y = 0.9433x + 1.6847 | 0.9962 |
| Set 3 (proteinase K before RNA extraction) | y = 0.9437x + 2.2062 | 0.9920 |
| Set 4 (proteinase K before bead) | y=0.9903x - 0.9962 | 0.7847 |

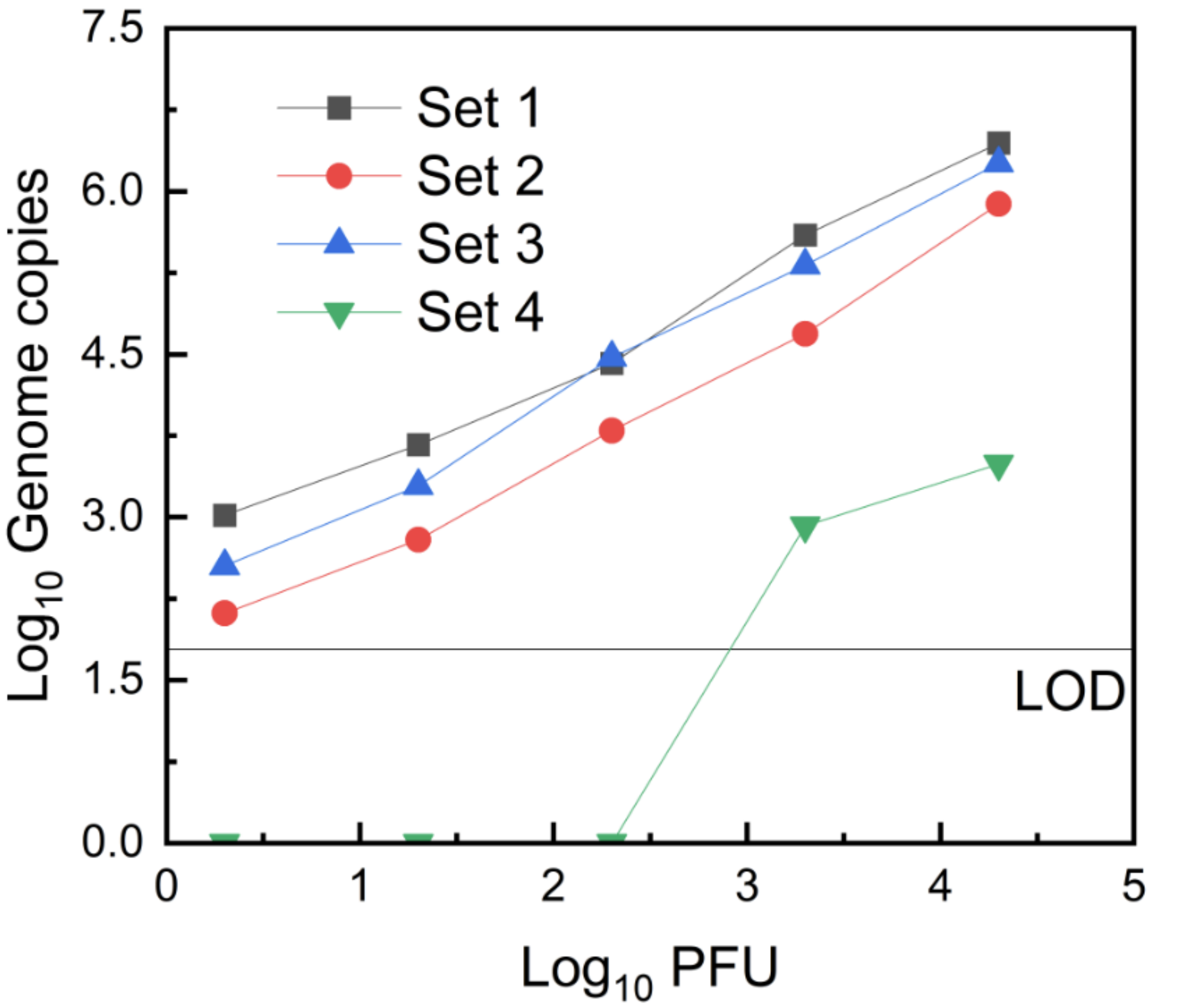


Figure S4: Relationships between PRCV genome copies and infectivity under different treatments

For Set 1, 2, and 3, good linear relationships existed between PRCV genome copies measured by N amplicon through one-step RT-qPCR and the actual infectivity measured by plaque assay. For set 1, even if the PRCV used was propagated from ST cells, some PRCV had damaged the protein structure during storage. Therefore, Set 2 had a slope closer to 1 and a smaller intercept, because PGM-MBs worked to capture virion with completed spike protein, and only a virus with the whole structure can be infectious. Set 3 used Proteinase K to hydrolyze a variety of peptide bonds. The fitting showed a similar slope to Set 2, and the slope was closer to 1, implying some proteins may inhibit the quantification slightly in Set 1. No genome was apparently damaged by Proteinase K considering the good linear relationships with the infectivity dose. Because of the different treatment method, the set1, set 2 and set 3 were significantly different(P value was less than 0.05 in between each Sets from both Dummy variable test and paired t test).

However, for set 4, no linear relationships were found. Proteinase K effectively damaged viral spike protein, therefore at a small PRCV infectivity dose, the amount of corresponding viral genome extracted from PGM-MBs binding virion was below detection limit. Only when the PRCV infectivity dose increased to 3.3 Log 10 and 4.3 Log10 we could detect N

amplicons, which may result from an inadequate dose of Proteinase K or inadequate incubation time for Proteinase K to damage virion proteins completely.

To conclude, 1) we tested the effectiveness of PGM-MBs assay in an infectivity dose from **2 PFU to 2*10^5 PFU**, which covered the dose of collected samples ( We generated PRCV aerosol using 2*10^6 PFU, and biosampler have collection efficiency around 1%); 2) we validated that PGM-MBs could effectively capture virion only with completed spike protein by comparing set 3 and set 4. ”

***RNase treatment***: For some experiments, aerosolized PRCV was collected in 20 mL PBS and then incubated with either RNase A/T1 mix (ThermoFisher) or PBS. Either 500 uL or 200 uL (as a negative control) of the virus-containing solution was added to new tubes. For the 500 uL aliquot, 50 uL of a 50-fold RNase A/T1 mix (ThermoFisher) was added to the tube. The tube was vortexed for 3 seconds and then incubated for 30 min at 37 $^{0}$C. Next, 50 uL of 50 fold SUPERase•In™ RNase Inhibitor (ThermoFisher) was added to the tube for a total of 600 uL. The tube was vortexed for 3 seconds and incubated for an additional 30 min at 37 $^{0}$C. For the tube containing the 200 uL aliquot of the virus-containing solution, which functioned as a negative control, 100 uL nuclease-free water (Corning) was added for a total of 300 uL. As mentioned above, the tube was vortexed for 3 seconds and then incubated.

***Validation of RNase assay***

The efficiency of RNase A/T1 by incubating (1) extracted PRCV RNA with 400 U RNase A/T1, (2) PCRV particles with 400 U RNase A/T1 or (3) PCRV particles with water for 30 min at 37 C. RNA was then re-purified and a portion of each reaction was subjected to one-step RT-qPCR to amplify the N gene. The CT value for the extracted PRCV RNA was below the limit of detection, indicating that the RNase A/T1 was indeed active. The CT values for RNA isolated from viruses after incubation with RNase A/T1 or water were 22.69 and 21.94, respectively. This implies that the RNase A/T1 solution did not penetrate and degrade viral RNA in the capsid.

**Section V: One-step RT-qPCR and long-range RT-qPCR**

***One-step RT-qPCR***: The SYBR-based one-step RT-qPCR reaction contained 3 uL of the sample with 5 µL 2X iTaq Universal SYBR Green 1-Step Reaction Mix (Bio-Rad Laboratories), 0.125 uL iScript Reverse Transcriptase (Bio-Rad Laboratories), 0.3 uL (10 uM) forward primer (5’-TCCTGGTGGTCTTTCAACCC-3’; IDT), 0.3 uL (10 uM) reverse primer (5’-CAGTTGGCACACCTTCGAGA-3’; IDT) and 1.275 uL nuclease-free water (Corning, NY, USA). The primers target the N gene. The 10 uL mixture was placed in one well of a 96-well plate (4306737, Applied Biosystems, USA) and reactions were run using a qPCR system (Quant Studio 3, Thermo Fisher Scientific, USA) with the following thermal cycle: 1 cycle (50 °C for 10 min and 95 °C for 1 min), 40 cycles (95 °C for 10 s, 60 °C for 30 s). Primers were expected to produce a 93-base pair amplicon. Each RNA sample was analyzed in triplicate. The resulting CT values from the above one-step RT-qPCR were then converted into corresponding genome copies concentration by fitting the CT into a standard curve. To create a standard curve each time, we used synthetic dsDNA (IDT) containing the N gene that would be detected by the above N primers (IDT). The sequence for the dsDNA is (5’-tgtacagaag gactaagtcc tggtggtctt tcaaccctga aactaatgca attctttgcg ttagtgcatt aggaagaagc tatgtgcttc ctctcgaagg tgtgccaact ggtgtcactc taactttgct ttcagg-3’). This dsDNA was serially

diluted ($10^1$ to $10^7$ copies/uL) and amplified at the same time and in the same plate containing extracted RNA samples. Each plate also contained three negative control wells, in which nuclease-free water was added instead of synthetic DNA.

***Long range RT-qPCR***: For the long-range RT-qPCR assays, 6 ul of each RNA extract was reverse-transcribed using 2uL PRCV-Reverse primer, 10uL M-MuLV Reaction Mix (NEW ENGLAND BioLabs) and 2uL M-MuLV Enzyme Mix (NEW ENGLAND BioLabs). The mixtures were incubated at 42 °C for 60 min, followed by 80 °C for 5 min in MyCycler Thermal Cycler (Bio-Rad Laboratories). Next, reactions were placed on ice for a short period. 3 uL of each reaction was incubated with 0.6 uL of 10 uM Forward primer (5’-TCCTGGTGGTCTTTCAACCC-3’), 0.6 uL of 10 uM Reverse primer ( 5’-TACCACCTCTTGCTCTGACCT-3’), 10 uL Sybr-green supermix(Bio-Rad Laboratories) and 5.8 uL nuclease-free water (Corning, NY, USA). The thermal condition of long-range PCR for PRCV cDNA was as follows: 1 cycle of 95 °C for 2 min, 25 cycles of 95 °C for 15 sec, 55 °C for 30 sec, and 72 °C for 90 sec using the Applied Biosystems QuantStudio 3 Real-Time PCR System. The following qPCR step was used to quantify DNA generated in the RT step after the amplification step. To ensure genome concentration was at the proper range for detection, DNA product from the long-range PCR step was diluted ten times with nuclease-free water (Corning, NY, USA) before use. QPCR reaction was initiated by mixing 2 µL of the diluted DNA, 7.5 µL of SYBR green Master Mix (Bio-Rad Laboratories), 0.375 uL of 10 µM forward primer, 0.375 uL of 10 µM reverse primer, and 4.75  uL nuclease-free water(Corning, NY, USA). The Applied Biosystems QuantStudio 3 Real-Time PCR System was used. The N gene copies were calculated using a standard curve (see one-step RT-qPCR above).

**Table S2.** PCR primers,sequences and thermal conditions

| One step | Primer(5’-3’) | Position | Thermal cycle conditions |
|---|---|---|---|
| One step RT-qPCR | F:TCCTGGTGGTCT TTCAACCC<br><br>R:CAGTTGGCACA CCTTCGAGA | F:25512 to 25531<br>R:25605 to 25586 | 1 cycle (50 °C for 10 min and 95 °C for 1 min), 40 cycles (95 °C for 10 s, 60 °C for 30 s) |
| Long range RT-PCR | Primer(5’-3’) | Position | Thermal cycle conditions |
| RT | R:TACCACCTCTTG CTCTGACCT | 26951 to 26931 | 42 °C for 60 min, followed by 80 °C for 5 min |
| Long range PCR | F:TCCTGGTGGTCT TTCAACCC<br><br>R:TACCACCTCTTG CTCTGACCT | 25512 to 26951 | 1 cycle of 95 °C for 2 min, 25 cycles of (95 °C for 15 s, 55 °C for 30 s, 72 °C for 90 s). |
| qPCR | F:TCCTGGTGGTCT TTCAACCC | F:25512 to 25531<br>R:25605 to 25586 | 95°C for 10 min; then 40 cycles of 95°C for 15 s and 60°C for 1 min |

| | | | |
|---|---|---|---|
| | R:CAGTTGGCACA CCTTCGAGA | | |
| Standard | Synthetic DNA sequence | position | Target gene |
| | tgtacagaag gactaagtcc tggtggtctt tcaaccctga aactaatgca attctttgcg ttagtgcatt aggaagaagc tatgtgcttc ctctcgaagg tgtgccaact ggtgtcactc taactttgct ttcagg | 25495-25630 | CDS: ABG89315.1 Name: nucleoprotein DQ811787.1 PRCV ISU-1 |

The standard curve had a slope between -3.8 to -3.2 and an intercept between 34 to 38. The PCR efficiency calculated from the slope was from 85% to 105%. Values $r^2$ for all calibration curves exceeded 0.98. The linear dynamic range (standard curve concentration) was between 10 to $10^7$ copies/μL. Cq variation at LOD for 12 repeats was 0.286 which equaled 3 copies/μL.

***Comparison of 1-step RT-qPCR with long-range PCR assay***

The N gene amplification results of both one-step RT-qPCR and long-range PCR reflect whether the genome is damaged or not. Theoretically, if the genome is randomly damaged, then long-range PCR is more likely to detect genomic damage due to the longer amplification fragments (1562 bp vs. 93 bp). However, if the genomic damage level is high and the target gene region for both one-step RT-qPCR and long-range PCR has been damaged, then the level of N gene amplicon reduction measured by one-step RT-qPCR and long-range PCR should be close to each other.

Table S3: Comparison of data from PCR assays fitted assuming first-order decay model

| RH Levels | Slopes of linear regression | | R-square | |
|---|---|---|---|---|
| | one step | long-range | one step | long-range |
| **RH 45-55** | **-0.015** | **-0.072** | **0.809** | **0.794** |
| RH55-65 | -0.011 | 0.003 | 0.754 | 0.020 |
| **RH65-75** | **-0.027** | **-0.021** | **0.921** | **0.662** |
| RH75-85 | -0.006 | -0.027 | 0.564 | 0.483 |

Table S3 used the average of N gene amplicon Removal ratio (Log $C/C_0$) as the axis and exposure time for different RHs as the x-axis, fitting data into a linear regression model (assuming RH led to first-order decay). For data collected in RH 45-55%, the R square for both one-step RT-qPCR and long-range PCR was around 0.8, making linear regression a comparable way. The slope derived from long-range PCR data was -0.072, around 5 times higher than the slope derived from one-step RT-qPCR, indicating higher genome removal ratio could be measured by long-range PCR over the same time, validating our conclusion that genome damage was observed at RH 45-55%.

For RH 65-75%, as R square for long-range PCR was below 0.8, we fitted the average of N gene amplicon Removal ratio (Log $C/C_0$) as y-axis and exposure time for different RHs as x-axis into 2 order polynomial curve (assuming RH led to second order decay, $y= Ax^2 + Bx + C$ ). It was complicated to distinguish whether long-range PCR measured more genome

degradation than one-step RT-qPCR because coefficients A and B caused a controversial trend. But by t-test comparison, we didn't find a significant difference between one-step RT-qPCR and long-range PCR data., which might be because targeted regions for both measurements' methods have been damaged. Though no significant difference between one-step RT-qPCR and long-range PCR was observed in RH 65-75%, we found data from RNase assay which reflected damage from both capsid and genome best estimated the infectivity loss measured by plaque assay as mentioned in section 3.3. The comparison between RNase assay and no RNase assay treatment (Figure 3C) showed no significant capsid damage in this RH level, thus genome damage should be an important factor.

| | coefficient 1(A) | | coefficient2(B) | | intercept (C) | | R square | |
|---|---|---|---|---|---|---|---|---|
| | one step | long-range | one step | long-range | one step | long-range | one step | long-range |
| RH 65-75 | 0.0005 | 0.0009 | -0.054 | -0.075 | -0.108 | -0.096 | 0.95 | 0.98 |

Table S4: Comparison of data from PCR assays fitted assuming second-order decay model

**Section VI: Statistical analysis (size bin has <0.25um and 0.1-0.25um as the lowest boundary)**

## Beads vs No beads (Paired test)

RH45-55
T-test for Unnamed: 1 and Unnamed: 2: p-value=nan
**T-test for Unnamed: 3 and Unnamed: 4: p-value=0.025**
T-test for Unnamed: 5 and Unnamed: 6: p-value=0.503
T-test for Unnamed: 7 and Unnamed: 8: p-value=0.515
T-test for Unnamed: 9 and Unnamed: 10: p-value=0.758

RH55-65
T-test for Unnamed: 1 and Unnamed: 2: p-value=nan
T-test for Unnamed: 3 and Unnamed: 4: p-value=0.133
**T-test for Unnamed: 5 and Unnamed: 6: p-value=0.0003**
Not enough data points for comparison between Unnamed: 7 and Unnamed: 8
T-test for Unnamed: 9 and Unnamed: 10: p-value=0.163

RH65-75
T-test for Unnamed: 1 and Unnamed: 2: p-value=nan
T-test for Unnamed: 3 and Unnamed: 4: p-value=0.578

T-test for Unnamed: 5 and Unnamed: 6: p-value=0.174
T-test for Unnamed: 7 and Unnamed: 8: p-value=0.617
T-test for Unnamed: 9 and Unnamed: 10: p-value=0.899

RH75-85
T-test for Unnamed: 1 and Unnamed: 2: p-value=nan
T-test for Unnamed: 3 and Unnamed: 4: p-value=0.005
T-test for Unnamed: 5 and Unnamed: 6: p-value=0.762
**T-test for Unnamed: 7 and Unnamed: 8: p-value=0.043**
T-test for Unnamed: 9 and Unnamed: 10: p-value=0.390

RNase vs No RNase (Paired Test)
RH 45-55
T-test for Unnamed: 1 and Unnamed: 2: p-value=nan
**T-test for Unnamed: 3 and Unnamed: 4: p-value=0.003**
T-test for Unnamed: 5 and Unnamed: 6: p-value=0.103
T-test for Unnamed: 7 and Unnamed: 8: p-value=0.316
T-test for Unnamed: 9 and Unnamed: 10: p-value=0.981

RH 55-65
T-test for Unnamed: 1 and Unnamed: 2: p-value=nan
T-test for Unnamed: 3 and Unnamed: 4: p-value=0.224
**T-test for Unnamed: 5 and Unnamed: 6: p-value=0.030**
**T-test for Unnamed: 7 and Unnamed: 8: p-value=0.004**
**T-test for Unnamed: 9 and Unnamed: 10: p-value=0.017**

RH 65-75
T-test for Unnamed: 1 and Unnamed: 2: p-value=nan
Wilcoxon signed-rank test for Unnamed: 3 and Unnamed: 4: p-value=0.125
T-test for Unnamed: 5 and Unnamed: 6: p-value=0.175
T-test for Unnamed: 7 and Unnamed: 8: p-value=0.216
**T-test for Unnamed: 9 and Unnamed: 10: p-value=0.002**

RH 75-85
T-test for Unnamed: 1 and Unnamed: 2: p-value=nan
T-test for Unnamed: 3 and Unnamed: 4: p-value=0.939
Wilcoxon signed-rank test for Unnamed: 5 and Unnamed: 6: p-value=0.062
**T-test for Unnamed: 7 and Unnamed: 8: p-value=0.019**
Wilcoxon signed-rank test for Unnamed: 9 and Unnamed: 10: p-value=0.62
T-test for Unnamed: 11 and Unnamed: 12: p-value=0.835

**For data from RH65-75%**
**Plaque assay vs RNase+long range (no significant difference)**
For Time Period 1, data is normally distributed. Paired T-test p-value: nan
For Time Period 2, data is normally distributed. Paired T-test p-value: 0.569
For Time Period 3, data is normally distributed. Paired T-test p-value: 0.338
For Time Period 4, data is not normally distributed. Wilcoxon Test p-value: 0.250

**For Time Period 5, data is normally distributed. Paired T-test p-value: 0.012**

**For data from RH65-75%**
**Plaque assay vs long-range(significant difference)**
For Time Period 1, data is normally distributed. Paired T-test p-value: nan
**For Time Period 2, data is normally distributed. Paired T-test p-value: 0.012**
**For Time Period 3, data is normally distributed. Paired T-test p-value: 0.032**
**For Time Period 4, data is normally distributed. Paired T-test p-value: 0.003**
**For Time Period 5, data is normally distributed. Paired T-test p-value: 0.004**

**For data from RH65-75%**
**Plaque assay vs one step(significant difference)**
For Time Period 1, data is normally distributed. Paired T-test p-value: nan
For Time Period 2, data is normally distributed. Paired T-test p-value: 0.062
For Time Period 3, data is normally distributed. Paired T-test p-value: **0.002**
For Time Period 4, data is normally distributed. Paired T-test p-value: **0.001**
For Time Period 5, data is normally distributed. Paired T-test p-value: **0.003**

Long-range PCR (use 0.1-0.25um as lowest boundary for F calculation)

RH45-55
T-test for Unnamed: 1 and Unnamed: 1: p-value=[nan nan]
T-test for Unnamed: 1 and Unnamed: 2: p-value=0.241
**T-test for Unnamed: 1 and Unnamed: 3: p-value=0.012**
**T-test for Unnamed: 1 and Unnamed: 4: p-value=0.034**
**T-test for Unnamed: 1 and Unnamed: 5: p-value=0.051**

RH55-65
T-test for Unnamed: 1 and Unnamed: 1: p-value=[nan nan]
T-test for Unnamed: 1 and Unnamed: 2: p-value=0.108
T-test for Unnamed: 1 and Unnamed: 3: p-value=0.092
T-test for Unnamed: 1 and Unnamed: 4: p-value=0.962
Wilcoxon signed-rank test for Unnamed: 1 and Unnamed: 5: p-value=0.5

RH65-75
T-test for Unnamed: 1 and Unnamed: 1: p-value=[nan nan]
Wilcoxon signed-rank test for Unnamed: 1 and Unnamed: 2: p-value=0.125
**T-test for Unnamed: 1 and Unnamed: 3: p-value=0.010**
**T-test for Unnamed: 1 and Unnamed: 4: p-value=0.004**
**T-test for Unnamed: 1 and Unnamed: 5: p-value=0.018**

RH75-85
T-test for Unnamed: 1 and Unnamed: 1: p-value=[nan nan]
T-test for Unnamed: 1 and Unnamed: 2: p-value=0.857
T-test for Unnamed: 1 and Unnamed: 3: p-value < 0.001 (No reduction)
T-test for Unnamed: 1 and Unnamed: 4: p-value=0.178
T-test for Unnamed: 1 and Unnamed: 5: p-value=0.188
T-test for Unnamed: 1 and Unnamed: 6: p-value=0.104

Long-range PCR (use <0.25um as the lowest size boundary for F calculation)

For Long Range 45-85:
RH 45-55
Column 1: Normally distributed. Paired T-test p-value: nan
Column 2: Normally distributed. Paired T-test p-value: 0.253
**Column 3: Normally distributed. Paired T-test p-value: 0.012**
**Column 4: Normally distributed. Paired T-test p-value: 0.033**
**Column 5: Normally distributed. Paired T-test p-value: 0.049**

RH 55-65
Column 1: Normally distributed. Paired T-test p-value: nan
Column 2: Normally distributed. Paired T-test p-value: 0.105
Column 3: Normally distributed. Paired T-test p-value: 0.080
Column 4: Normally distributed. Paired T-test p-value: 0.773
Column 5: Normally distributed. Paired T-test p-value: 0.479

RH 65-75

Column 1, data is normally distributed. Paired T-test p-value: nan
Column 2, data is not normally distributed. Wilcoxon Test p-value: 0.125
**Column 3, data is normally distributed. Paired T-test p-value: 0.010**
**Column 4, data is normally distributed. Paired T-test p-value: 0.004**
**Column 5, data is normally distributed. Paired T-test p-value: 0.020**

RH 75-85:
Column 1: Normally distributed. Paired T-test p-value: nan
Column 2: Normally distributed. Paired T-test p-value: 0.881
**Column 3: Normally distributed. Paired T-test p-value < 0.001**
Column 4: Normally distributed. Paired T-test p-value: 0.219
Column 5: Normally distributed. Paired T-test p-value: 0.177
Column 6: Normally distributed. Paired T-test p-value: 0.088

**Section VII: Propagation of error in calculating $F(t_{exp})$**

The first step towards calculating $F(t_{exp})$ is the determination of the deposition coefficient ($k$; arising from gravitational settling, Brownian diffusion, and wall losses) from the SMPS+OPS data based on three repeat experiments. Polynomial regression (2nd Degree) was used to represent the $k$ values (based on the average of the three repeats) as a function of particle diameter $D_p$, as follows:

$$k = [0.1147 \pm 0.0060] \cdot (\log_{10} D_p)^2 - [0.5869 \pm 0.0304] \cdot \log_{10} D_p + [0.7528 \pm 0.0371], (6)$$

where each coefficient is presented as the best-fit coefficient ($C_i$) and the standard error ($\sigma_{C_i}$) associated with the coefficient at a significance level (α) = 0.05. The average ($k_a$), lower-bound ($k_l$), and upper-bound ($k_u$) of the $k$ values for sizes 250, 500, 1000, and 2500 nm were determined by assuming the coefficients (i.e., 0th, 1st, and 2nd order coefficients) to be $C_i$, $C_i + \sigma_{C_i}$, and $C_i - \sigma_{C_i}$, respectively. As $\sigma_{C_i}$, was the standard error of the best-fit coefficient at α = 0.05, the probability of $k$ being between $k_l$ and $k_u$ was 95%. The average of $k_l$, $k_u$, and $k_a$ were used to calculate the mean ($k_i$) and standard deviation ($\sigma_{k_i}$) of $k_i$ for each of the five particle sizes ($i$).

Ensuing this, we calculated the remaining percentage of suspended aerosols $[S_i(t_{exp})]$ at exposure time ($t_{exp}$) following nebulization, was evaluated assuming first-order deposition losses for each of the four sizes, $i$ (i.e., 0.25, 0.5, 1, and 2.5 μm) using a mass-balance approach with the following equation:

$$S_i(t_{exp}) = 100 \cdot e^{-k_i \cdot t_{exp}} \quad (7)$$

Propagation of error was used to calculate the standard deviation of $S_i(t_{inc})$ using:[4]

$$\sigma_{S_i(t_{exp})} = |S_i(t_{exp})| \cdot t_{exp} \cdot \sigma_{k_i} \quad (8)$$

For size bins 250 – 500 nm, 500 – 1000 nm, and 1000 – 2500 nm, the remaining percentage of suspended aerosols in each of these size bins was calculated using the mean of the percentage of suspended aerosols in the lower and upper size bounds of the size bins, as depicted in the following example:

$$S_{0.25-0.5}\,(t_{exp}) = \frac{S_{0.25}(t_{exp}) + S_{0.5}(t_{exp})}{2} \quad (9)$$

The propagation of error in equation 4 was evaluated using:

$$\sigma_{S_{0.25-0.5}(t_{exp})} = |S_{0.25-0.5}\,(t_{exp})| \cdot \sqrt{\left(\sigma_{S_{0.25}(t_{exp})}\right)^2 + \left(\sigma_{S_{0.5}(t_{exp})}\right)^2} \quad (10)$$

For the smallest (< 0.25 μm) and largest size-bins (> 2.5 μm), only the upper bound (0.25 μm) and lower bound (>2.5 μm) of these bins were used to make the above calculations, respectively.

Finally, $F(t_{exp})$ were calculated by adding the sum of the product of the percentage of viruses (based on the data from the cascade impactor filters) and the percentage of aerosols that remain suspended in the air for the five size-bins, $j$ [$\epsilon$ $(1-5)$], at $t_{exp}$, using:

$$F(t_{exp}) = \sum_{j=1}^{5} S_j(t_{exp}) \cdot A_j \ , \tag{11}$$

where $A_j$ is the particle distribution percentage in size bin $j$, based on the N gene amplicon levels. The $A_j$ values at different RH levels are illustrated in Figure S4.

The standard deviation of $F(t_{exp})$ was evaluated using the propagation of error as follows:

$$\sigma_{F(t_{exp})} = \sqrt{\sum_{j=1}^{5}\left(\left|S_j(t_{exp}) \cdot A_j\right| \cdot \sqrt{\left(\frac{\sigma_{S_j(t_{exp})}}{S_j(t_{exp})}\right)^2 + \left(\frac{\sigma_{A_j}}{A_j}\right)^2}\ \right)^2} \tag{12}$$

The $F(t_{exp})$ values with standard deviations at different are provided in Tables S4, S5, S6, and S7 for 45-55%, 55-65%, 65-75%, and 75-85% RH levels, respectively.

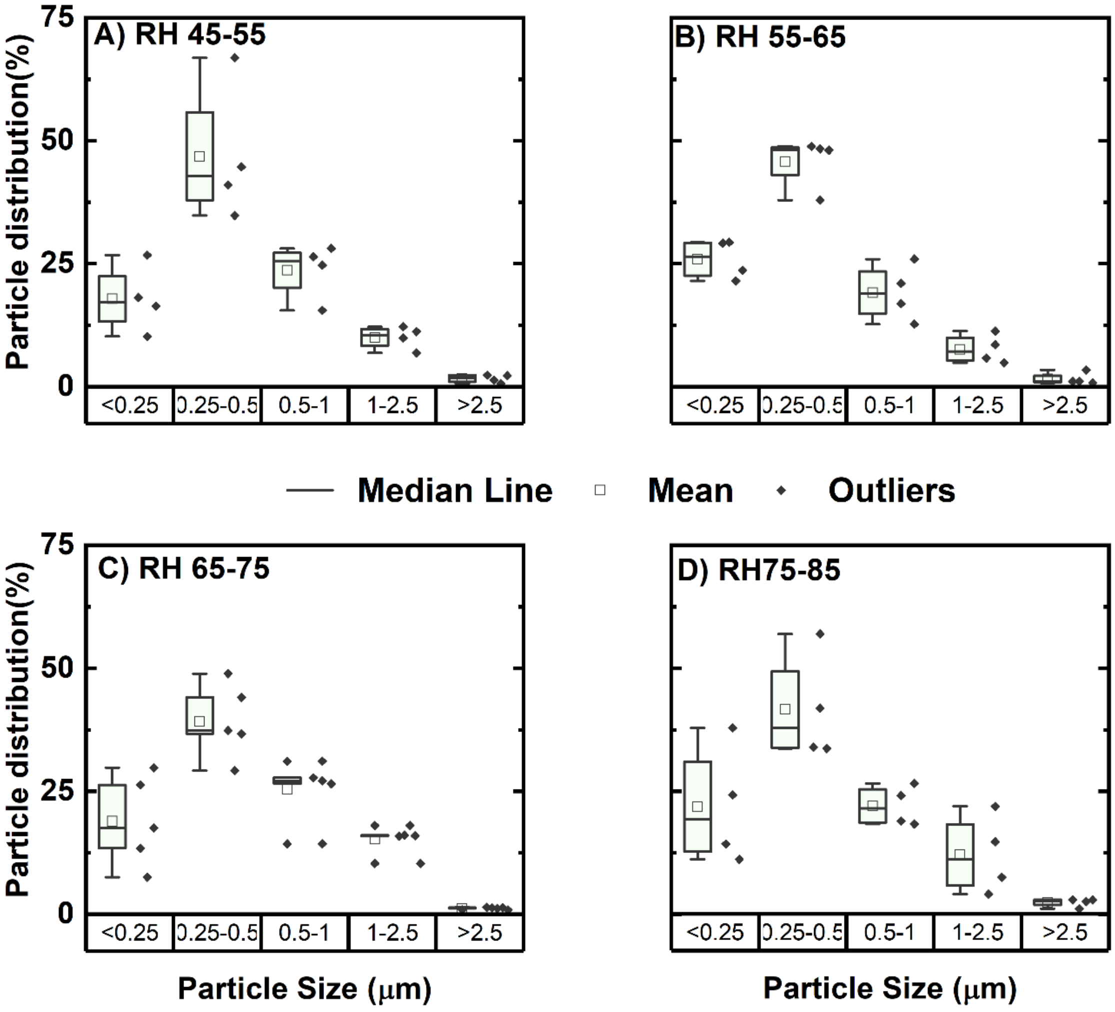

**Figure S5: Effects of various humidities on PRCV particle distribution as measured by N gene amplicon levels via one-step RT-qPCR after cascade impactor collection and sonication.**
**Data in Figure S5 were used to calculate corresponding F factors as described in Section 3.1 in the manuscript.**

Table S5: $F(t_{exp})$ values and their standard deviation at 45 – 55% RH.

| RH45-55 | | Exposure time ($t_{exp}$; minutes) | | | | | |
|---|---|---|---|---|---|---|---|
| | | 5 | 10 | 20 | 30 | 40 | 60 |
| aerosol size(μm) | distribution percentage % ($A_j$) | The remaining percentage of aerosols suspended in the air at $t_{exp}$ for each size bin [%; $S_j(t_{exp})$] | | | | | |
| <0.25 | 17.85 | 97.54 | 95.13 | 90.51 | 86.1 | 81.91 | 74.14 |
| 0.25-0.5 | 46.82 | 97.71 | 95.46 | 91.14 | 87 | 83.06 | 75.7 |
| 0.5-1 | 23.67 | 93.19 | 87.07 | 76.58 | 68 | 60.94 | 50.2 |
| 1-2.5 | 10.02 | 77.3 | 61.01 | 40.23 | 28.21 | 20.66 | 11.91 |
| >2.5 | 1.64 | 66.08 | 43.66 | 19.06 | 8.32 | 3.63 | 0.69 |
| $F(t_{inc})$ (%) | | 94.05 | 89.11 | 81.30 | 75.16 | 70.06 | 61.76 |
| S.D. | | 16.79 | 16.00 | 15.03 | 14.37 | 13.85 | 13.08 |

Column 1: Five size bins of particles segregated by cascade impactor.
Column 2: Aerosol particle distribution as measured by N gene for different size bins.
Columns 3-8: Percentage of remaining aerosol not deposited at five size bins for corresponding exposure times from 5 minutes to 60 minutes, calculated by applying mean deposition coefficient and time into equation (5).
Row 9: Summary of percentage of remaining aerosols at overall sizes for corresponding exposure times from 5 minutes to 60 minutes, calculated by applying aerosol particle distribution and into equation (6).
Row 10: Standard deviation of each $F(t_{exp})$

Table S6: $F(t_{exp})$values and their standard deviation at 55 – 65% RH.

| RH55-65 | | Exposure time ($t_{exp}$; minutes) | | | | | |
|---|---|---|---|---|---|---|---|
| | | 5 | 10 | 20 | 30 | 40 | 60 |
| aerosol size (μm) | distribution percentage % ($A_j$) | The remaining percentage of aerosols suspended in the air at $t_{exp}$ for each size bin [%; $S_j(t_{exp})$] | | | | | |
| <0.25 | 25.9 | 97.54 | 95.13 | 90.51 | 86.1 | 81.91 | 74.14 |
| 0.25-0.5 | 45.78 | 97.71 | 95.46 | 91.14 | 87 | 83.06 | 75.7 |
| 0.5-1 | 19.12 | 93.19 | 87.07 | 76.58 | 68 | 60.94 | 50.2 |
| 1-2.5 | 7.61 | 77.3 | 61.01 | 40.23 | 28.21 | 20.66 | 11.91 |
| >2.5 | 1.58 | 66.08 | 43.66 | 19.06 | 8.32 | 3.63 | 0.69 |
| $F(t_{inc})$ | | 94.74 | 90.32 | 83.17 | 77.41 | 72.52 | 64.37 |
| S.D. | | 8.66 | 8.27 | 7.78 | 7.56 | 7.51 | 7.71 |

Table S7: $F(t_{exp})$values and their standard deviation at 65 – 75% RH.

| RH65-75 | | Exposure time ($t_{exp}$; minutes) | | | | | |
|---|---|---|---|---|---|---|---|
| | | 5 | 10 | 20 | 30 | 40 | 60 |
| aerosol size (μm) | distribution percentage % ($A_j$) | The remaining percentage of aerosols suspended in the air at $t_{exp}$ for each size bin [%; $S_j(t_{exp})$] | | | | | |
| <0.25 | 18.91% | 97.54 | 95.13 | 90.51 | 86.1 | 81.91 | 74.14 |
| 0.25-0.5 | 39.25% | 97.71 | 95.46 | 91.14 | 87 | 83.06 | 75.7 |
| 0.5-1 | 25.36% | 93.19 | 87.07 | 76.58 | 68 | 60.94 | 50.2 |
| 1-2.5 | 15.26% | 77.3 | 61.01 | 40.23 | 28.21 | 20.66 | 11.91 |
| >2.5 | 1.22% | 66.08 | 43.66 | 19.06 | 8.32 | 3.63 | 0.69 |
| $F(t_{inc})$ | | 93.03 | 87.38 | 78.68 | 72.08 | 66.74 | 58.29 |
| S.D. | | 13.23 | 12.78 | 12.05 | 11.48 | 11.05 | 10.43 |

Table S8: $F(t_{exp})$ values and their standard deviation at 75 – 85% RH.

| RH75-85 | | Exposure time ($t_{exp}$; minutes) | | | | | |
|---|---|---|---|---|---|---|---|
| | | 5 | 10 | 20 | 30 | 40 | 60 |
| aerosol size (µm) | distribution percentage % ($A_j$) | The remaining percentage of aerosols suspended in the air at $t_{exp}$ for each size bin [%; $S_j(t_{exp})$] | | | | | |
| <0.25 | 21.9 | 97.54 | 95.13 | 90.51 | 86.1 | 81.91 | 74.14 |
| 0.25-0.5 | 41.62 | 97.71 | 95.46 | 91.14 | 87 | 83.06 | 75.7 |
| 0.5-1 | 22.01 | 93.19 | 87.07 | 76.58 | 68 | 60.94 | 50.2 |
| 1-2.5 | 12.07 | 77.3 | 61.01 | 40.23 | 28.21 | 20.66 | 11.91 |
| >2.5 | 2.4 | 66.08 | 43.66 | 19.06 | 8.32 | 3.63 | 0.69 |
| $F(t_{inc})$ | | 93.46 | 88.14 | 79.92 | 73.64 | 68.50 | 60.25 |
| S.D. | | 17.44 | 16.65 | 15.55 | 14.77 | 14.16 | 13.24 |

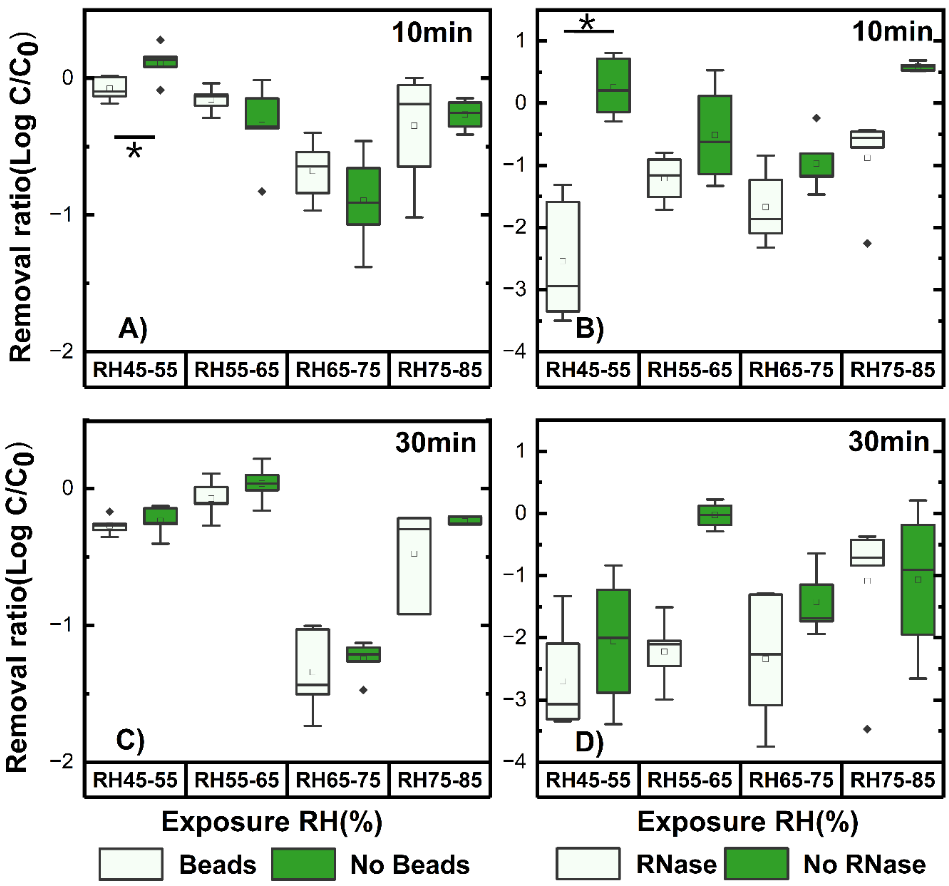

**Figure S6: Effects of various humidities on spike protein as measured by N gene amplicon levels via one-step RT-qPCR at certain time periods, 10 min(A) and 30 min (C). Effects of various humidities on capsid integrity and genome integrity as measured by N gene amplicon levels via long-range PCR at certain time periods, 10 min(B) and 30 min (D).**

Figure S6 compared Beads treated vs no beads treated (A and C), RNase treated vs no RNase treated (B and D), and long-range PCR (B and D, No RNase) for each RH level under the same time periods. No continuous damage on spike protein or capsid was observed at any RH levels. Genome damage levels were found to be significant between RH 45-55 and 65-75, RH 55-65 and 75-85, and RH 65-75 and 75-85 at 10 minutes, in addition, significant differences were found between RH 55-65 and 65-75 at 30minutes, indicating that with increase of time exposure, higher genome removal ratio occurred at RH 45-55% and RH65-75%. This was because, at 10 minutes, genome was less removed at RH 45-55% compared with RH65-75% but at 10 minutes, the removal ratio had no significant differences, indicating genome was removed more at RH 45-55% than at RH 65-75% during 10-30 minutes. Similarly, the removal ratio had no significant differences between RH 55-65% and RH 65-75% at 10 minutes but genome was less removed at RH 55-65% than at RH 65-75% at 30 minutes, indicating genome was removed more at RH 65-75% than at RH 55-65% during 10-30 minutes.

**Table S9:** Coefficient of variance (CV) for t=0 at different RH levels under each treatment assays

| CV=STD/Ave | Beads | No beads | RNase | No RNase |
|---|---|---|---|---|
| RH 45-55% | 11.00% | 6.64% | 8.96% | 2.68% |
| RH 55-65% | 22.08% | 68.79% | 9.90% | 25.42% |
| RH 65-75% | 30.63% | 30.65% | 37.28% | 47.16% |
| RH 75-855 | 9.69% | 12.88% | 16.35% | 8.40% |

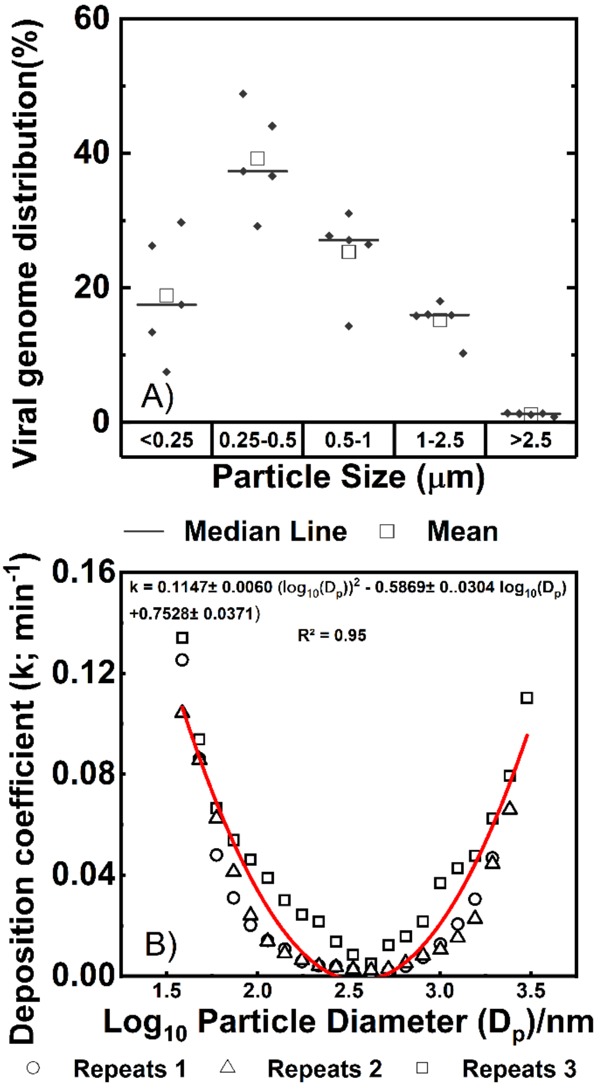


**Figure S7:** Particle distribution and deposition coefficient after nebulization. For each time point, $2\times10^6$ PFU of PRCV were aerosolized by nebulization. Viruses were released into the closed chamber and then collected immediately. (A)A cascade impactor was used to collect aerosolized virus for 15 minutes. Quartz filters inside each stage of the cascade impactor were collected, submerged in PBS, and sonicated for 1 hour. RNA was extracted from the PBS solution post-sonication for each size range. An aliquot of RNA from each sample was analyzed for the NP gene by using one-step RT-qPCR, and genome copies were calculated by using a standard curve. Particle percentage distribution was calculated by dividing genome copies of each size range into overall genome copies, which was the summary of genome copies for all size ranges. (B) Particle deposition coefficient distribution was determined by combining

SMPS and OPS data. In this case, PBS (with no virus) was aerosolized by nebulization. HEPA-filtered air was used to dilute aerosolized PBS, allowing PBS particles to be counted by SMPS (10-378 nm) and OPS (300-10,000 nm). Coefficient distribution was then calculated by a first-order reaction equation with a known air change rate.

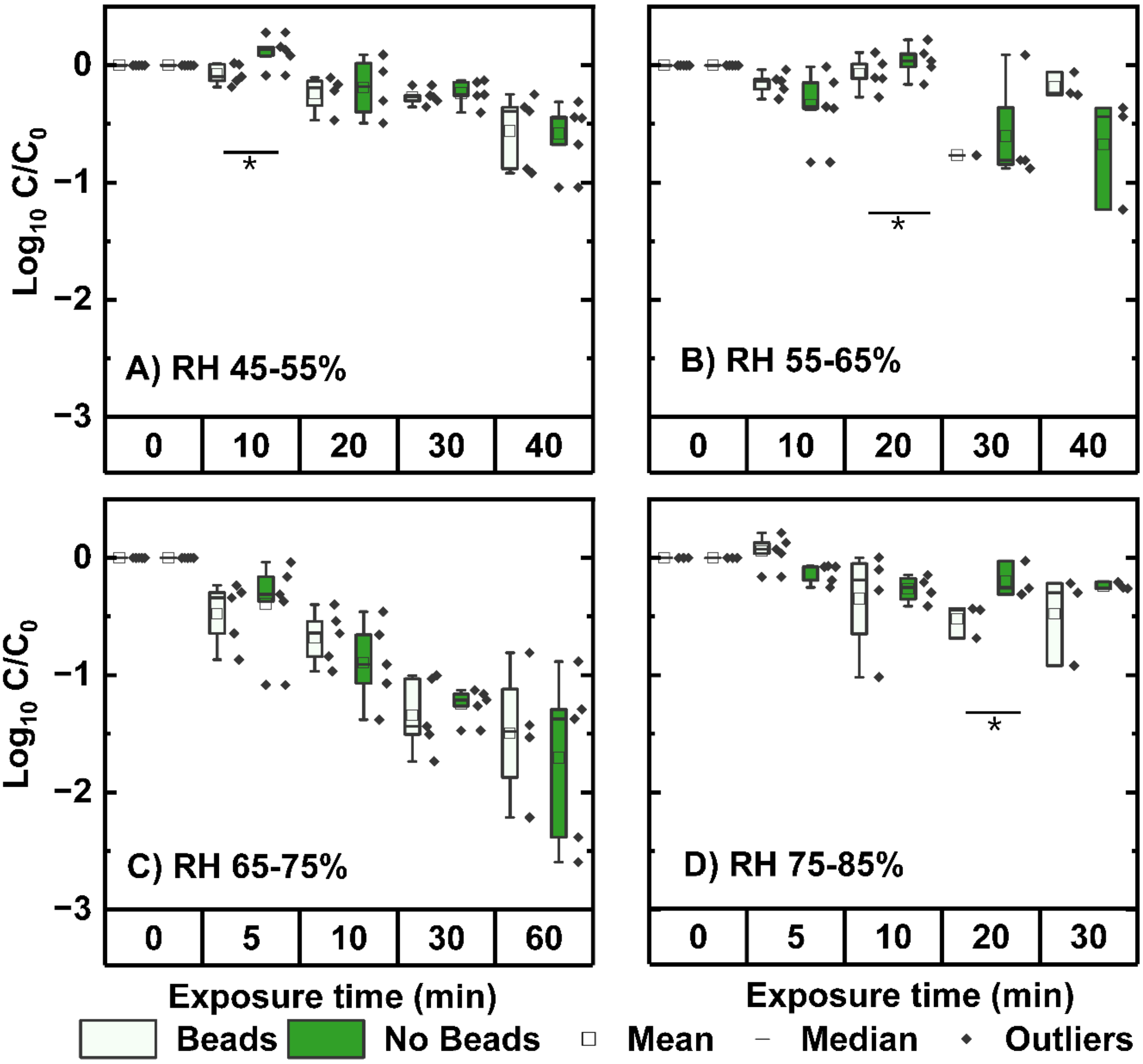


**Figure S8:** Effects of humidity on aerosolized PRCV binding to PGM-MB as measured by N gene levels. $2\times10^6$ PFU of PRCV was aerosolized by nebulization for each experiment. Once the virus was in the nebulizer, the RH was adjusted by the connected humidifier. The RH used was (a) 45-55% (b) 55-65% (c) 65-75% or (d) 75-85%. Then, viruses were released into the closed chamber and incubated for times indicated on the x-axis. A biosampler was used to

collect aerosolized virus for 15 minutes. A portion of each sample was either not incubated or incubated with PGM-MB beads at room temperature for 30 minutes. Beads were washed three times in PBS. Then, RNA was extracted from the beads and subjected to one-step RT-qPCR to amplify the N gene, and genome copies were calculated. Data were expressed as $Log_{10}$ $C/C_0$ where $C_0$ was the average value of independent experiments from samples at 0 min for each different RH condition. *Data for RH55-65%, 30min were below LOD.

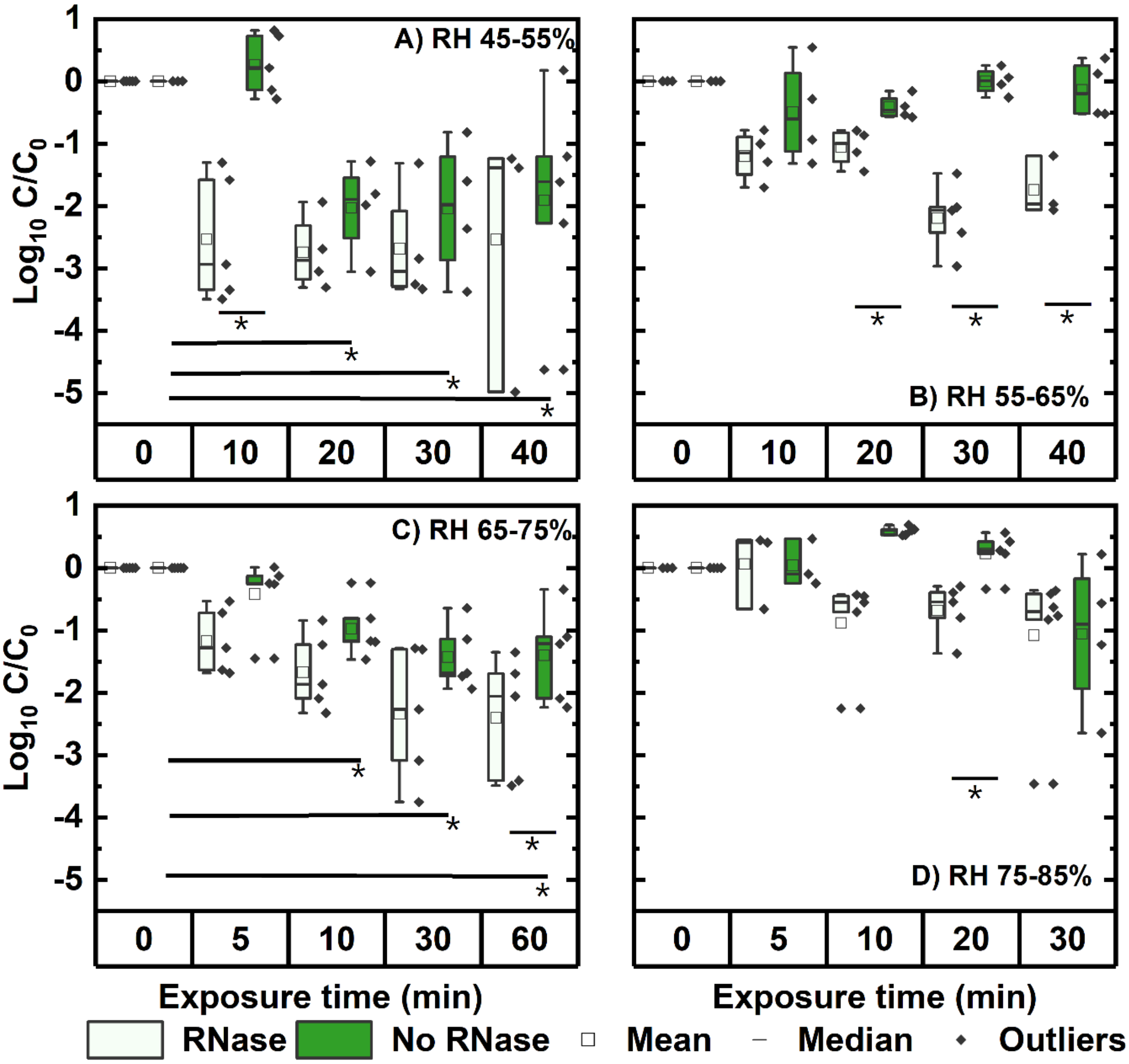


**Figure S9:** Effects of humidity on capsid stability of aerosolized PRCV. Viral samples were nebulized, exposed to different RH conditions, and collected in the same way as described in Figure S3. Then, a portion of each sample was either not treated or treated with RNase A/T1

and RNase inhibitor at 37C. Then, RNA was extracted from each sample and subjected to one round of reverse transcription. Next, purified cDNA was amplified by using a two-step strategy: 25 amplification cycles of the N gene region (long-range PCR) and 40 amplification cycles using nested primers within the N gene. Genome copies were calculated as described in Section V.

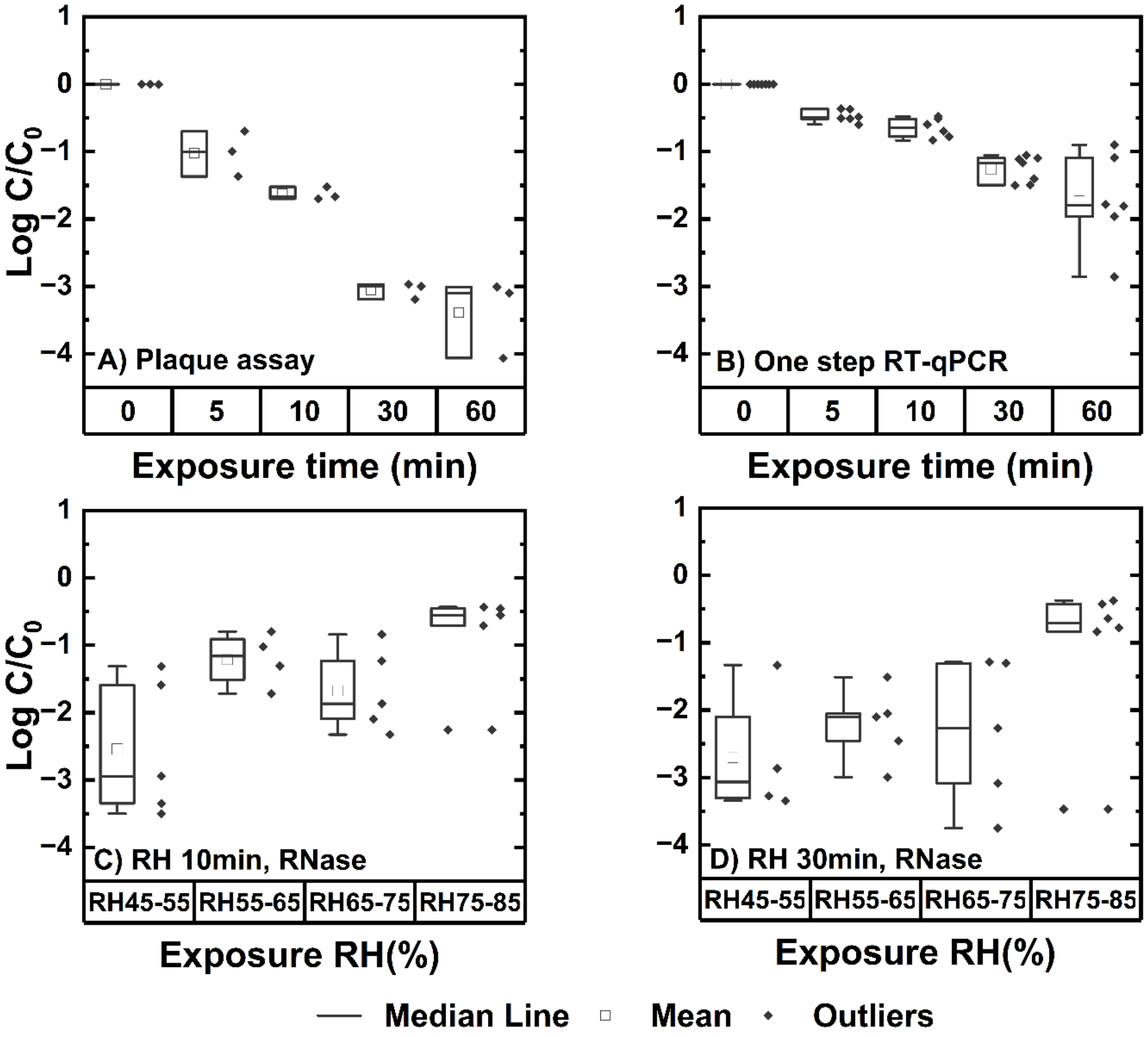


**Figure S10:** Effects of RH 65-75% on aerosolized PRCV as measured by (A) Viral infectivity and plaque forming assays (conducted using $2 \times 10^6$PFU of PRCV once and $2 \times 10^7$PFU twice), (B) N gene amplicon levels via one-step RT-qPCR. Effects of various humidities on aerosolized PRCV as measured by N gene amplicon levels via long-range RT-qPCR with RNase A/T1 treatment at (C) 10 minutes and (D) 30 minutes RH exposure times.

Data were expressed as $C/C_0$ where $C_0$ was the average value of independent experiments from samples at 0 min at corresponding RH levels. Data had been corrected from $Log_{10}$ $C/C_0$ to $Log_{10}$ $C/C_0$ – $Log_{10}$ $F(t_{exp})$ to exclude deposition effects.

## References


(1) *Antibiotic-Antimycotic (100X) from Thermofisher*. https://www.thermofisher.com/order/catalog/product/15240062.

(2) Batterman, S. Review and Extension of CO2-Based Methods to Determine Ventilation Rates with Application to School Classrooms. *Int. J. Environ. Res. Public. Health* **2017**, *14* (2), 145. https://doi.org/10.3390/ijerph14020145.

(3) Subramanian, P. S. G.; Puthussery, J. V.; Mao, Y.; Salana, S.; Nguyen, T. H.; Newell, T.; Verma, V. Influence of Human Activities and Occupancy on the Emission of Indoor Particles from Respiratory and Nonrespiratory Sources. *ACS EST Air* **2024**, *1* (5), 386–396. https://doi.org/10.1021/acsestair.3c00088.

(4) *Propagation_of_uncertainty*. https://en.wikipedia.org/wiki/Propagation_of_uncertainty. https://en.wikipedia.org/wiki/Propagation_of_uncertainty (accessed 2024-06-19).
